# Prognosis of immune checkpoint inhibitor-induced myasthenia gravis: a single center experience and systematic review

**DOI:** 10.3389/fneur.2024.1372861

**Published:** 2024-04-03

**Authors:** Yuhui Qin, Siyuan Chen, Qian Gui, Teng Zhang, Yanan Li, Zhijuan Du, Yahui Lv, Xiangyu Du, Yi Hu, Zhefeng Liu

**Affiliations:** ^1^Department of Medical Oncology, Senior Department of Oncology, The Fifth Medical Center of PLA General Hospital, Beijing, China; ^2^Medical School of Chinese PLA, Beijing, China; ^3^School of Medicine, Jianghan University, Wuhan, China; ^4^Department of Oncology, The 983rd Hospital of Joint Logistic Support Force of PLA, Tianjin, China

**Keywords:** myasthenia gravis, immune checkpoint inhibitors, immunotherapy, immune-related adverse events, neurotoxicity

## Abstract

**Background:**

Immune checkpoint inhibitors (ICI)-induced myasthenia gravis (MG) is an uncommon but potentially fatal neurotoxicity. We aim to help physicians familiarize themselves with the clinical characteristics of ICI-induced MG, facilitating early diagnosis and prompt intervention.

**Methods:**

We searched the Chinese People’s Liberation Army General Hospital medical record system from January 2017 to August 2023 for patients diagnosed with ICI-induced MG. We systematically reviewed the literature until August 2023 to identify all similar patients. We collected clinical information on these patients.

**Results:**

110 patients were identified, 9 from our institution and 101 from case reports. In our institution, Median age was 66 years (range: 49–79 years). 6 were males. The most common was lung cancer (*n* = 4). All patients had no previous history of MG and received PD-1 or PD-L1 inhibitors. The median time from ICI initiation to first MG symptoms was 4 weeks (range: 2–15 weeks). ICIs were discontinued in all patients. Most patients initially received high-dose corticosteroids, and their symptoms improved. Some patients are discharged with corticosteroids maintenance therapy. In addition, 55 patients (50%) with concomitant myositis and/or myocarditis and MG-induced mortality were more common in the myositis and/or myocarditis group (10.9% vs. 34.5%, *p* = 0.016). Overlap of myositis with MG (OR = 3.148, *p* = 0.009) and anti-AChR antibody positivity (OR = 3.364, *p* = 0.005) were both significantly associated with poor outcomes.

**Conclusion:**

Our study reveals the prognosis of ICI-induced MG and suggests that myositis and/or myocarditis are severe comorbidities of ICI-induced MG, emphasizing the importance of early diagnosis and clinical intervention.

## 1 Introduction

Immune checkpoint inhibitors (ICI) mainly include anti-programmed cell death protein 1 (PD-1) antibodies (e.g., nivolumab, pembrolizumab, tislelizumab, etc.), anti-programmed Cell Death Protein-Ligand 1 (PD-L1) antibodies (e.g., atezolizumab, durvalumab, etc.), and anti-cytotoxic T Lymphocyte-Antigen 4 (CTLA-4) antibodies (e.g., ipilimumab, etc.). Typically, tumors directly or indirectly reduce the intensity and extent of the immune response through immune checkpoints to maintain the self-tolerance of tumor cells in their surrounding normal tissues and to evade immune detection. Specifically, targeted binding of anti-PD1, anti-PDL1, and anti-CTLA4 antibodies enhances the anti-tumor immune response and accelerates host-mediated destruction of malignant cells by promoting immune surveillance (1). ICI have been widely used to treat non-small cell lung cancer (NSCLC), metastatic melanoma, renal cell carcinoma, and other tumors (2, 3). Nonetheless, the incidence of adverse drug reactions is on the rise, especially the non-specific characteristics of neurological Immune-induced adverse events (NirAEs), which are challenging to recognize and treat (4).

Immune-induced adverse events (IrAEs) have the potential to affect any organ system. However, the gastrointestinal tract, endocrine glands, skin, liver, and lungs are the most frequently involved, with a lower incidence of NirAEs. However, it is associated with higher mortality (5). The most commonly reported symptom of NirAEs is headache. They may also involve the peripheral and central nervous system (6). Myasthenia gravis (MG) is an autoimmune disorder impacting the neuromuscular junction, commonly identified by symptoms like ptosis and diplopia. In severe cases, it may involve the respiratory and masticatory muscles, leading to dyspnea and dysphagia. Acetylcholine receptor antibodies (AChR-Abs) and anti-muscle specific kinase antibodies (anti-MuSK Abs) are highly diagnosis-specific, with detection rates of around 85 and 10%, respectively (7, 8). ICI-induced MG is more difficult to diagnose and is often combined with myositis and/or myocarditis, with a rapid progression of the disease, often leading to patient death (9). Early recognition and effective clinical management are crucial. We reviewed our institution’s database and searched the literature for relevant case reports to summarize the prognostic and clinical characteristics of 110 patients with MG in the context of receiving ICI.

## 2 Materials and methods

### 2.1 Patients

**Figure 1:**
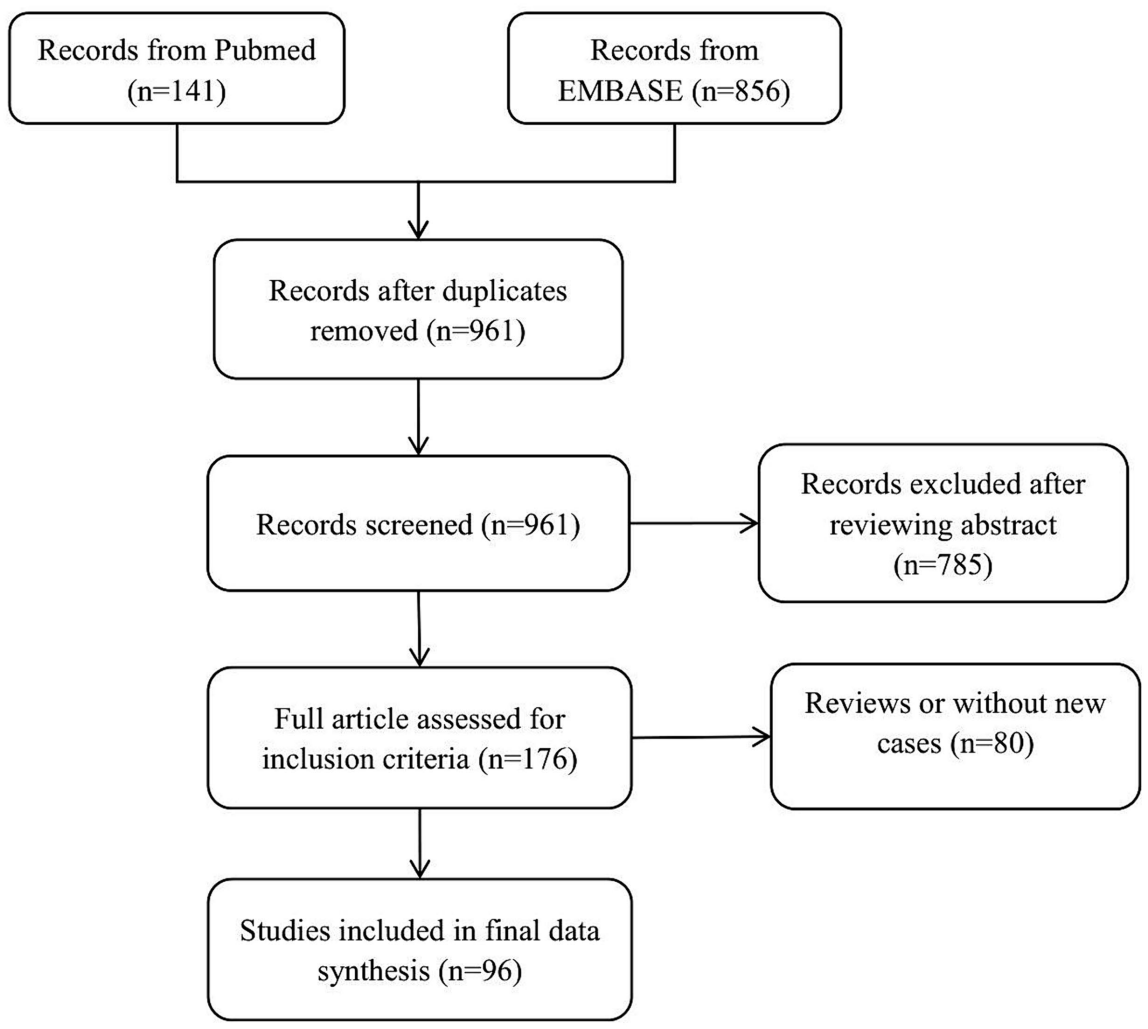
Study selection flowchart.

Patients diagnosed with ICI-induced MG at PLAGH between January 2017 and August 2023 constituted the study cohort. We conducted a comprehensive search on PubMed and Embase for case reports, series, and observational studies documenting cancer and MG patients undergoing ICI until August 2023 without imposing language or research design limitations. The search strategy and terms can be found in Supplementary File 1. Diagnostic criteria for ICI-induced MG are described in Supplementary File 2. Figure 1 illustrates the flow chart for screening case reports. Additionally, the quality appraisal of the reported cases from the literature is detailed in Supplementary Table 3. Then, the complete texts of the chosen articles were examined. We manually looked through the references of the included articles. Each patient had a comprehensive clinical profile.

### 2.2 Methods

We retrieved patient demographic and baseline characteristic data from PLAGH and literature-identified patients. Data from our institution and case reports were divided into two groups: MG alone and MG concomitant myositis and/or myocarditis. The two groups’ clinical and diagnostic characterization, management, and outcomes were evaluated and compared.

### 2.3 Statistical analysis

The study underwent statistical analysis using SPSS 26 and GraphPad Prism 8.0.2. For categorical data assessment, frequencies and percentages were employed, while medians and ranges described continuous data. The significance of categorical variables was compared between the two groups using the χ^2^ test. Continuous variables were assessed using the Mann–Whitney U test. Univariate binary logistic regression models were employed to calculate odds ratios (ORs) for the association between specific clinical or demographic variables and the risk of adverse events in MG patients. Additionally, a multivariate binary logistic regression model was utilized to examine the components significantly linked to negative results. All tests were two-sided, and statistical differences were deemed significant if *p* < 0.05.

## 3 Results

Out of 961 unique articles identified in the literature, 96 publications, detailing 101 patients, met the inclusion criteria, with an additional 9 patients identified from PLAGH. Consequently, our final analysis encompassed a total of 110 patients; Median age was 72 years (range: 30–90 years), 67 were males. The most common type of cancer was melanoma (*n* = 34), followed by lung cancer (*n* = 31). 12 patients had a previous history of MG. Most patients received PD-1 inhibitors. 55 patients were diagnosed with MG combined with myositis and/or myocarditis. Among them, myositis was also diagnosed in 20 and myocarditis in 25; 10 had the triad of MG/myositis/myocarditis. For patients with the triad of MG/myositis/myocarditis, all patients had a rapid onset of illness after receiving the first or second cycle of ICI therapy. Almost all patients received steroids, and the remaining common treatments included intravenous immunoglobulin (IVIG; *n* = 9), acetylcholinesterase inhibitors (*n* = 5), and mechanical ventilation (*n* = 5). Eventually, 7 patients reported death.

**Figure 2:**
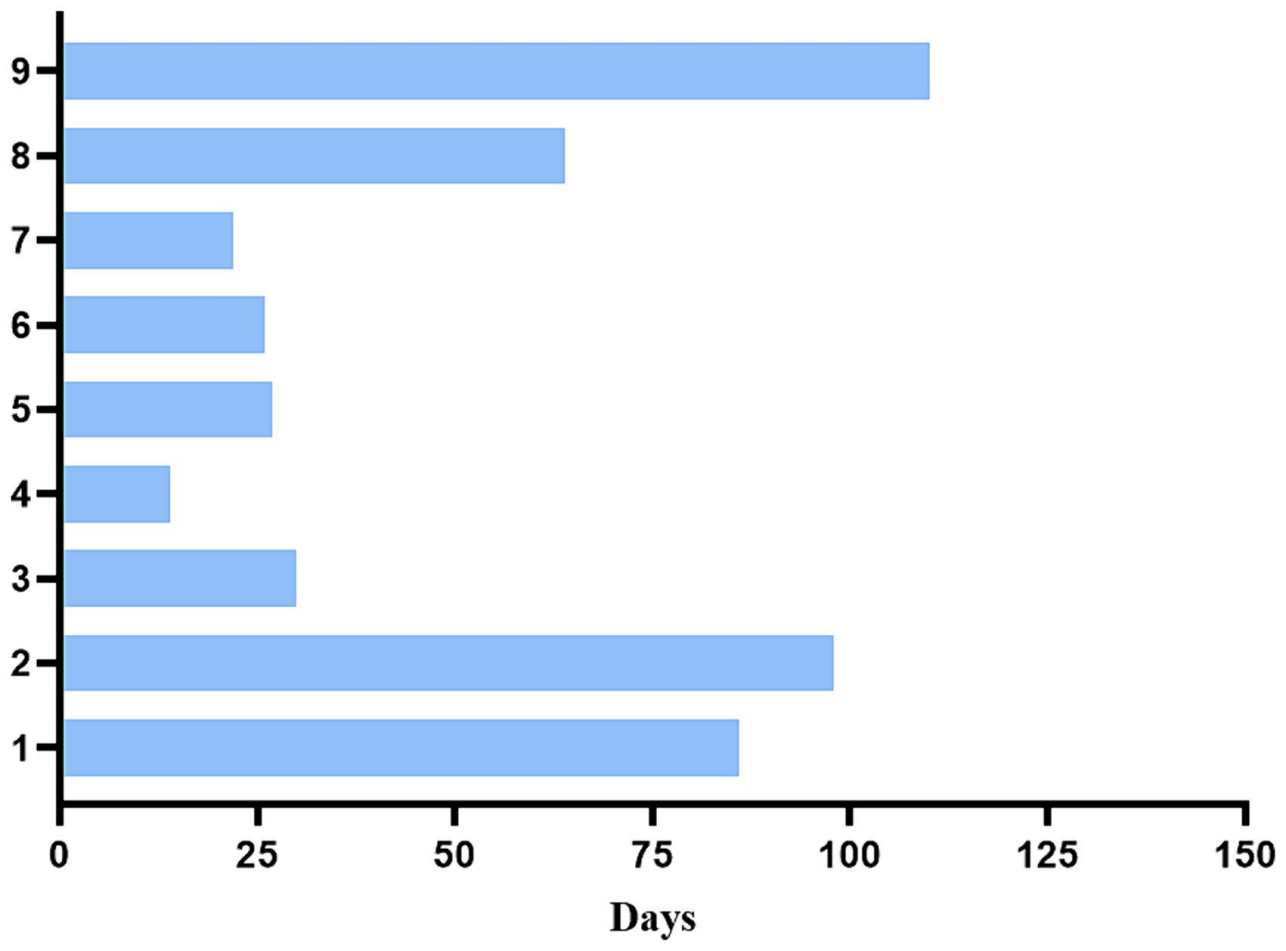
Time start of immune checkpoint inhibitors to clinical myasthenia gravis.

9 patients from PLAGH were diagnosed with ICI-induced MG. The clinical data of the patients were summarized in Table 1. Their median age was 66 years (range: 49–79 years). 6 patients were male. The most common type of cancer was lung cancer (n = 4), followed by esophageal cancer (*n* = 2). None of the 9 patients had a previous history of MG, and all received PD-1 or PD-L1 inhibitors. 4 patients were treated with ICI combined with targeted therapy, three with ICI combined with chemotherapy, one with ICI combined with HDAC inhibitor, and one with ICI alone therapy. The PDL1-expression level on tumor cells was available for 5 patients: < 1% for 3%, 1%–49% for 1, and >50% for 1. Of these patients, 5 patients developed symptoms immediately after their first or second ICI. The median time from ICI initiation to first MG symptoms was 4 weeks (range: 2–15 weeks) (Figure 2).

**Table 1:** The demographic and clinical information of the patients from PLAGH.

ID	Sex/ age	Type of cancer	Type of ICI	Past MG	Treatment cycle	Onset (weeks)	Other irAEs	Clinical presentation	AChR status	CPK	Treatment	MG outcome	Death
1	M/79	Bladder cancer	Durvalumab	NO	4	12	Myocarditis	Ptosis, Diplopia, Dysphagia, Neck weakness, Limb weakness	Positive	380	Prednisone (120 mg) + Pyridostigmine (15 mg) + rituximab (0.5 g)	Improvement	NO
2	M/67	NSCLC (adeno)	Pembrolizumab	NO	5	14	Myocarditis, liver injury, thyroid injury	Limb weakness, Myalgias	Negative	2,208	Prednisone (120 mg)	Improvement	NO
3	F/62	Esophagus cancer	Treiprilizumab	NO	2	4	Myocarditis, myositis	Ptosis, Diplopia, Limb weakness, Generalized weakness	Negative	NI	Prednisone (120 mg) + Pyridostigmine (60 mg) + IVIG (0.4 g/kg/d)	Deterioration	Cancer progression
4	M/76	NSCLC (squamous)	Tislelizumab	NO	1	2	Myocarditis, liver injury	Ptosis, Neck weakness, Generalized weakness, dyspnea	Positive	1,368	Prednisone (120 mg) + MMF (500 mg) + IVIG (0.4 g/kg/d)	Improvement	NO
5	M/66	Colorectal cancer	Treiprilizumab	NO	2	4	Myocarditis	Ptosis, Limb weakness	Negative	9,994	Prednisone (240 mg) + IVIG(0.4 g/kg/d)	Improvement	NO
6	F/64	Esophagus cancer	Carirelizumab	NO	1	4	Myocarditis, myositis	Limb weakness, dyspnea	Positive	2,023	Methylprednisolone (500 mg) + IVIG (0.4 g/kg/d)	Improvement	Cancer progression
7	F/75	NSCLC (adeno)	Carirelizumab	NO	1	3	Myositis	Myalgias	Positive	2,460	Methylprednisolone (500 mg) + IVIG (0.4 g/kg/d)	Improvement	Cancer progression
8	M/49	Colorectal cancer	Carirelizumab	NO	3	9	NO	Ptosis, Diplopia, Limb weakness	Negative	2,792	Prednisone (120 mg) + Pyridostigmine (60 mg) + IVIG (0.4 g/kg/d)	Improvement	NO
9	M/58	NSCLC (adeno)	Cindilimumab	NO	5	15	NO	Limb weakness	Negative	NI	Prednisone (120 mg) + Pyridostigmine (60 mg)	Improvement	NO

M, male; F, female; NSCLC, non-small-cell lung cancer; AChR, acetylcholine receptor; MG, myasthenia gravis; CPK, creatine phosphokinase; IVIG, intravenous immunoglobulin; MMF, mycophenolate mofetil; Nl, within normal range.

The most common symptoms at first presentation were ptosis (*n* = 5) and dysphagia (*n* = 5), and the rest of the frequent symptoms included dyspnea and limb weakness and excluded exacerbation of symptoms due to progression of the primary tumor. Myocarditis was also diagnosed in 6 patients, myositis was diagnosed in 3 patients, and liver injury in 2 patients. 4 patients were found to have positive anti-AChR antibodies. 7 patients were detected with elevated creatine phosphokinase (CPK; median 2,208 IU/L, range: 380–9,994 IU/L). 2 patients had electromyography showing myogenic injury, and one patient had a muscle biopsy showing disseminated myofibrillar necrosis with type II myofibrillar atrophy. Computed tomography or magnetic resonance imaging was performed in 7 patients to rule out brain metastases or acute intracranial events. 3 patients had abnormal electrocardiograms on admission, demonstrating third-degree atrioventricular and right bundle branch blocks.

ICIs were stopped for all patients. They all received corticosteroids, 5 with acetylcholinesterase inhibitors, 6 with immunoglobulins, and the rest of the treatments included rituximab and mycophenolate mofetil (MMF). Overall, MG symptoms improved in 8 and worsened in 1 patient. 3 patients reported death, and the cause of death was systemic organ failure, all due to the tumor. Six patients are currently alive.

### 3.1 MG alone and comparison with the MG combined myositis or/and myocarditis

We grouped a total of 110 patients who were hospitalized and reported in the literature. The control group was patients with MG alone (without comorbid myositis and/or myocarditis), and the experimental group was patients with MG comorbid with myositis and/or myocarditis. We compared the clinical characteristics and prognosis of the two groups as shown in Table 2. Our results showed no statistically significant differences between the two groups in terms of age, gender, previous MG, and type of ICI. The specific information is shown in Table 2 and Supplementary Tables 4, 5. Secondly, regarding the clinical features at the onset, the myositis and/or myocarditis group developed MG-induced symptoms earlier after ICI treatment than patients with MG alone (*p* < 0.001). Anti-AchR antibody positivity was more common in the myositis and/or myocarditis group (*p* = 0.029). Regarding treatment, there were no statistically significant differences between the two groups in the use of corticosteroids, intravenous immunoglobulin, and plasma exchange. A relatively higher proportion of patients with MG alone were treated with anticholinesterase inhibitors (72.7% vs. 40.0%, *p* = 0.001). In addition, because patients with myositis and/or myocarditis were more severely ill and more likely to have myasthenia gravis-associated respiratory failure, more patients received mechanical ventilation compared to patients with MG alone (16.4% vs. 41.8%, *p* = 0.006). Finally, in terms of prognosis, symptoms being more challenging to treat in the myositis and/or myocarditis group, MG-induced mortality was higher in the myositis and/or myocarditis group (10.9% vs. 34.5%). In contrast, MG alone responded better to treatment, with more deaths due to cancer progression (12.7% vs. 5.5%). Our study suggests that myositis and/or myocarditis are common comorbidities of ICI-induced MG, which severely affects the prognosis of patients.

**Table 2:** Clinical features and prognosis of ICI-related MG combined with myositis/myocarditis.

	Myositis/myocarditis(−) *N* = 55; *n*(%)	Myositis/myocarditis(+) *N* = 55; *n*(%)	*P*-value
Mean age (years)	74 (67–78)	69 (64–77)	0.061
Male gender	33 (60)	34 (61.8)	1.000
Type of cancer			0.016
Melanoma	24 (43.6)	10 (18.2)	
Lung cancer	15 (27.3)	16 (29.1)	
Kidney cancer	3 (5.5)	9 (16.4)	
Others	13 (23.6)	20 (36.4)	
Type of ICI			0.178
PD(L)-1	49 (89.1)	48 (87.3)	
CTLA-4	4 (7.3)	1 (1.8)	
PD-1 + CTLA-4	2 (3.6)	6 (10.9)	
Past MG	9 (16.4)	3 (5.5)	0.124
Treatment cycle			<0.001
1	9 (16.4)	30 (54.5)	
2	20 (36.4)	20 (36.4)	
≥3	26 (47.3)	5 (9.1)	
Onset			<0.001
≤4	16 (29.1)	45 (81.8)	
>4	39 (70.9)	10 (18.2)	
**Clinical presentation**			
Ptosis	33 (60)	35 (63.6)	0.854
Diplopia	29 (52.7)	18 (32.7)	0.053
Dyspnea	15 (27.3)	17 (30.9)	0.834
Dysphagia	19 (34.5)	13.6 (23.6)	0.294
Limb weakness	18 (32.7)	21 (38.2)	0.690
Anti-AChR	29 (52.7)	41 (74.5)	0.029
CPK (IU/L)	2,155 (1,542–5,266)	3,922 (1,586–9,246)	0.132
**Treatment**			
Corticosteroids	50 (90.9)	54 (98.2)	0.206
Acetylcholinesterase inhibitors	40 (72.7)	22 (40.0)	0.001
IVIG	28 (50.9)	34 (61.8)	0.336
Plasmapheresis	17 (30.9)	20 (36.4)	0.687
Ventilation	9 (16.4)	23 (41.8)	0.006
MG outcome			0.007
Deterioration	8 (14.5)	22 (40.0)	
Improvement	42 (76.4)	31 (56.4)	
Complete resolution	5 (9.1)	2 (3.6)	
Death			0.016
MG complications	6 (10.9)	19 (34.5)	
Cancer progression	7 (12.7)	3 (5.5)	
Others	8 (14.5)	4 (7.3)	

Values are mean ± SD, *n* (%), or median (interquartile range). ICI, immune checkpoint inhibitor; PD-1, Programmed cell death 1; PD-L1, Programmed cell death-Ligand 1; CTLA-4, Cytotoxic T-Lymphocyte antigen 4, MG, myasthenia gravis; CPK, creatine phosphokinase; IVIG, intravenous immunoglobulin.

### 3.2 Factors influencing the prognosis of patients with ICI-induced MG

The results of univariate binary logistic regression analysis of the association between clinical characteristics and ICI-induced MG prognosis are shown in Table 3. Shorter cycles of ICI treatment (OR = 4.929, *p* = 0.003) and earlier onset of symptoms (OR = 2.501, *p* = 0.023) were negatively associated with ICI-induced MG adverse outcomes. The overlap of myositis with MG (OR = 3.148, *p* = 0.009) and Anti-AChR antibody positivity (OR = 3.364, *p* = 0.005) was significantly associated with poor ICI-induced MG outcomes. However, we did not find a correlation between the degree of CPK elevation and adverse disease outcomes. We incorporated variables such as cancer type, type of ICI, treatment cycle, onset, Anti-AChR antibody status, combined with myositis, and treatments into the multivariate binary logistic regression model. The results of the multivariate analysis indicated a negative association between the combined with myositis and Anti-AChR antibody positivity with outcomes in ICI-induced MG.

**Table 3:** Univariate and multivariate analyses for factors affecting the prognosis of patients with ICI-related MG (*n* = 110).

Variables	Univariate analyses		Multivariate analyses	
	OR(95%CI)	*P*-value	OR(95%CI)	*P*-value
Age, years (≤70/>70)	0.700 (0.326–1.503)	0.361		
Gender (Female/Male)	1.060 (0.488–2.301)	0.883		
**Type of cancer**				
Melanoma	0.516 (0.195–1.366)	0.183	0.59 (0.196–1.773)	0.348
Lung cancer	0.341 (0.121–0.960)	0.042	0.246 (0.072–0.838)	0.025
Kidney cancer	1.167 (0.307–4.441)	0.821	1.081 (0.252–4.639)	0.916
Others				
**Type of ICI**				
PD(L)-1	1.273 (0.288–5.628)	0.751		
CTLA-4	1.111 (0.112–10.986)	0.928		
PD-1 + CTLA-4				
Past MG (yes/no)	1.390 (0.418–4.619)	0.591		
**Treatment cycle**				
1	4.929 (1.714–14.174)	0.003	1.846 (0.377–9.216)	0.445
2	2.534 (0.887–7.239)	0.083	1.04 (0.249–4.342)	0.957
≥3				
Onset (≤4/>4)	2.501 (1.137–5.502)	0.023	0.86 (0.261–2.829)	0.804
Combined with myositis (yes/no)	3.148 (1.340–7.397)	0.009	4.415 (1.413–12.160)	0.001
Combined with myocarditis (yes/no)	1.195 (0.533–2.682)	0.665		
CPK (≤2,000/>2,000 IU/L)	2.500 (0.813–7.689)	0.110		
Anti-AChR (positive/negative)	3.364 (1.429–7.916)	0.005	2.91 (1.033–8.196)	0.043
**Treatment (yes/no)**				
Corticosteroids	1.525 (0.267–8.701)	0.635		
Acetylcholinesterase inhibitors	1.465 (0.679–3.158)	0.330		
IVIG	2.347 (1.067–5.159)	0.034	2.094 (0.843–5.257)	0.115
Plasmapheresis	0.874 (0.391–1.950)	0.741		
Ventilation	0.885 (0.384–2.042)	0.775		

ICI, immune checkpoint inhibitor; PD-1, Programmed cell death 1; PD-L1, Programmed cell death-Ligand 1; CTLA-4, Cytotoxic T-Lymphocyte antigen 4, MG, myasthenia gravis; CPK, creatine phosphokinase; IVIG, intravenous immunoglobulin.

### 3.3 Rechallenge with ICI

There is insufficient literature to provide evidence suggesting the risk of ICI re-initiation after NirAEs. Our review of case reports identified nine patients who were retreated with the same or a different ICI after initial MG remission due to a lack of effective alternative therapy to manage their advanced malignancies (melanoma [*n* = 6], lung cancer [*n* = 1], ovarian cancer [*n* = 1], and uterine carcinosarcoma [*n* = 1]). 6 patients received the same initial medication (anti-PD1), and the 2 patients were switched from pembrolizumab to nivolumab. Another patient was switched from nivolumab combined with ipilimumab to nivolumab monotherapy. After the first MG remission, all patients continued prophylaxis with corticosteroids, pyridostigmine, and IVIG. Recurrence of MG was seen in only 1 patient, and the irAE was seen in 2 cases (including 1 thyroiditis and 1 hepatitis). 7 patients ultimately had partial or complete tumor responses, and one patient died due to rapid tumor progression. Efficacy evaluation was not reported in another case.

## 4 Discussion

We searched medical records and reviewed the literature for ICI-induced MG. NirAEs are less common, with incidence rates of only 1%–5% (10), with an incidence of 0.2% for irAE-MG. Compared with other irAEs, the mortality rates are high (11). According to meta-analyses of clinical trials, the incidence of any grade of irAE was 66 and 72% for PD-1/PD-L1 inhibitors and CTLA-4 inhibitors, respectively. The incidence of severe irAE was 14 and 24% for PD-1/PD-L1 inhibitors and CTLA-4 inhibitors, respectively (12, 13), and 0.3%–1.3% were lethal (14).

There were no differences in the clinical features and prognosis of ICI-induced MG between patients with previous MG episodes and those experiencing new-onset disease. The actual incidence of MG episodes following ICI in patients with a previous MG diagnosis remains unknown, primarily due to the absence of pertinent cohort studies. In addition, conventional studies often exclude individuals with a history of autoimmunity, posing challenges in evaluating the impact of ICI in substantial subject cohorts. The precise mechanism through which ICI induces MG remains currently unknown. It remains questionable whether patients with new-onset MG have subclinical autoimmunity that manifests only after exposure to ICI. Studies have demonstrated that CTLA-4 knockout mice can spontaneously develop MG (15). Furthermore, specific CTLA-4 genetic variants predispose individuals to MG, particularly in Caucasian and East Asian populations (16, 17). Overexpression of PD-1 is linked to favorable outcomes in autoimmune diseases, as it facilitates CD8 T cell depletion; however, PD-1 inhibitors may worsen symptoms in individuals with pre-existing MG (18). Currently, additional studies are required to validate these findings.

A review of 110 cases supports that ICI-induced MG is a life-threatening irAE that rapidly deteriorates shortly after ICI initiation. 32 patients presented with dyspnea requiring mechanical ventilation. In addition, we found that ICI-induced MG was often concurrent with myocarditis and/or myositis. 110 patients were grouped into a control group without other irAEs and an experimental group with other irAEs, such as myocarditis and myositis. The results showed a higher mortality in the MG combined myositis/myocarditis group. Our study supports that ICI-mediated MG and its overlapping syndrome occur early after the initiation of therapy and are associated with significant mortality.

ICI-induced MG have a 57%–83% positive rate of anti-AchR antibodies, in addition to the common neostigmine test and ice test, and some patients may be combined with hyper CPK and even positivity of myositis-associated antibodies, which often suggests that simultaneous combination of ICI-induced myositis may be possible. In our study, 70 patients were positive for anti-AchR antibodies, in addition to Anti-AchR, Anti-Striated muscle antibodies (antititin, anti-heart muscle, and anti-skeletal muscle autoantibodies) have been identified in both ICI-induced MG and myositis (19, 20), While myositis-specific autoantibodies tend to be negative (21–23). Müller-Jensen et al. studies have demonstrated that the presence of ICI-induced neuromuscular disease in cancer patients with 80 and 88% sensitivity and specificity, respectively, for the detection of these autoantibodies. Neuromuscular autoantibodies may serve as viable markers for the diagnosis and potential prediction of life-threatening ICI-induced neuromuscular diseases (24).

7 patients were treated with PD-1 combination with CTLA-4 inhibitor, six of whom had comorbid myositis and/or myocarditis, suggesting that the combination of the two drugs may be associated with a higher incidence of irAE. The reported incidence and distribution of irAE may vary by drug type, PD-1, PD-L1, CTLA-4, or combination. The incidence of serious irAE is as high as 27% with anti-CTLA4 compared to 16% with anti-PD1 and may increase to 55% when both therapies are used concurrently (25). Meanwhile, the incidence and severity of irAE are higher when CTLA-4 inhibitors are used alone or in combination with PD-1 or PD-L1 drugs, such as ipilimumab and nivolumab, regardless of the treatment of the primary tumor (26–28). A meta-analysis showed that the risk of irAE was elevated in solid tumors when ICI was added to chemotherapy, regardless of the drug (and tumor type) used (29).

Myocarditis and NirAEs are life-threatening initial irAEs, and clinicians should be more cautious in evaluating ICI rechallenge in these patients. Therefore, more evidence is needed to assess the safety and efficacy of rechallenge. Pembrolizumab and nivolumab were the most frequently utilized ICI in various studies, serving as both primary and secondary therapies. Moreover, these agents share similar three-dimensional structures and effector mechanisms; nevertheless, pembrolizumab exhibits a higher affinity for recombinant human PD-1 compared to nivolumab (30). Kan et al. demonstrated an enhanced response in four melanoma patients treated initially with nivolumab and subsequently with pembrolizumab (31). In a cohort encompassing diverse cancer types, predominantly melanoma and lung cancer, Simonaggio et al. reported a modest improvement in overall response rate with anti-PD-1 or anti-PD-L1 inhibitors. This finding suggests that, within the context of successful and well-tolerated primary treatment, which may support re-treatment with the same drug or drug group, the initial response to ICI treatment could serve as a crucial predictor of re-challenge efficacy (32, 33). Currently, prior or combined radiotherapy, chemotherapy, or targeted therapy is regarded as a promising strategy to enhance the effectiveness of both primary and secondary immunotherapy. Research by Niki et al., Watanabe et al., and Xu et al. has indicated that patients who exhibited a positive response to a second ICI received intermittent treatment with radiotherapy, chemotherapy, or targeted therapy, particularly in patients with NSCLC (34–36).

9 patients in MG symptomatic remission could be rechallenged with ICI without compromising ICI efficacy at a reduced dose of steroids, prophylaxis with pyridostigmine, and/or IVIG after carefully evaluating available treatment options. There needs to be adequate literature on the impact of rechallenge on survival, irAE recurrence, and the incidence of new irAE. Despite the risk of recurrent irAE, initiating ICI after discontinuation for prior MG may be partially safe with careful clinical monitoring and aggressive prevention. An investigation on the safety of rechallenge revealed that the recurrence rate of irAE varied based on the organ involved in the initial irAE. It was lower in patients re-initiated with the same ICI drug or ICI combination compared to those re-initiated with a different ICI drug or ICI combination (*p* = 0.02). The median duration of ICI discontinuation to re-initiation and the severity of the initial irAE were not predictive of recurrent irAE after ICI re-initiation (37). Previous literature shows that periodic irAE is not as severe as the first one and that patients continue to respond to medication after the episode (38).

As shown in the current study, ICI-induced MG is often combined with myositis and/or myocarditis and is significantly associated with poor prognosis (39). We observed that patients with combined myositis and/or myocarditis had a significantly shorter time to disease onset after the first or second dose of ICI treatment than patients with MG alone. This trend toward early onset of disease may indicate that flares and/or progression in the myositis and/or myocarditis group were more rapid in patients with poorer outcomes. Although ICI toxicity may occur at any time during treatment, Our findings align with prior studies indicating that fatal ICI toxicity often manifests early, potentially within 4 weeks of treatment initiation or shortly after the initial dose (40). Other studies have confirmed troponin as a possible predictor and have used elevated creatinine and decreased urea and hemoglobin as early biomarkers in dying patients (41).

ICI-related MG hardly resolves spontaneously and requires immediate hospitalization once detected. In terms of treatment, patients require immediate discontinuation of ICI. The current study found that this group of patients responds poorly to acetylcholinesterase inhibitor therapy unless symptoms are nonprogressive and mild, but high-dose methylprednisolone shock combined with IVIG or plasma exchange has been recommended as an important therapeutic measure in studies of ICI-related MG treatment. It can be used to alleviate myasthenia gravis crisis. In addition, a combination of immunomodulators such as MMF, rituximab, and infliximab may be considered in addition to the above treatment options. Patients with overlap syndrome have a potentially fatal risk of rapid progression to serious adverse events, and the main interventions currently available for this group of patients include recognition of the triad, airway support, administration of high-dose methylprednisolone, and ensuring early involvement of the multidisciplinary team.

The fact that ICI-induced MG is a relatively uncommon complication limits the study. There are no established diagnostic standards for diagnosing MG, myositis, and myocarditis. In particular, MG can be diagnosed in some patients who are negative for AChR antibodies. The results of our study will help clinicians familiarize themselves with the clinical features of ICI-induced MG. They will help them to make an early diagnosis of the disease and intervene promptly. In addition, our findings offer a safety signal for patients with no available alternatives in advanced stages that may help clinicians balance each patient’s advantages and disadvantages.

## 5 Conclusion

Although ICI-induced MG is rare, it often involves the respiratory muscles, leading to dyspnea and high mortality. Moreover, most patients remain symptomatic after treatment, seriously affecting their quality of life. In addition, half of the patients with ICI-induced MG often have a combination of myositis and/or myocarditis, which contributes to the rapid deterioration of the patient’s disease. Therefore, it is essential to recognize this possible complication as early as possible in patients treated with ICI, and the necessity of a multidisciplinary strategy and multimodal active treatment should also be recognized.

## Data availability statement

The original contributions presented in the study are included in the article/Supplementary material, further inquiries can be directed to the corresponding authors.

## Author contributions

YQ: Conceptualization, Data curation, Formal analysis, Methodology, Software, Supervision, Writing – original draft, Writing – review & editing. SC: Conceptualization, Data curation, Formal analysis, Methodology, Software, Supervision, Writing – original draft, Writing – review & editing. QG: Conceptualization, Data curation, Formal analysis, Methodology, Software, Supervision, Writing – original draft, Writing – review & editing. TZ: Conceptualization, Data curation, Formal analysis, Writing – original draft, Writing – review & editing. YLi: Conceptualization, Data curation, Formal analysis, Writing – original draft, Writing – review & editing. ZD: Data curation, Formal analysis, Writing – original draft, Writing – review & editing. YLv: Data curation, Formal analysis, Writing – original draft, Writing – review & editing. XD: Data curation, Formal analysis, Writing – original draft, Writing – review & editing. YH: Conceptualization, Methodology, Supervision, Writing – original draft, Writing – review & editing. ZL: Conceptualization, Methodology, Supervision, Writing – original draft, Writing – review & editing.
