# Supplementary material for: Prognosis of immune checkpoint inhibitor-induced myasthenia gravis: a single center experience and systematic review
Source: Front Neurol. 2024 Apr 3;15:1372861. doi: 10.3389/fneur.2024.1372861 (PMC11022771; doi:10.3389/fneur.2024.1372861)
Supplement: Supplementary file 1 [file Table_1.DOCX]

**Search Strategy**

一、Pubmed

1."Immune Checkpoint Inhibitors"[MeSH Terms]

2.'Checkpoint Inhibitors, Immune'[Title/Abstract] OR 'Immune Checkpoint Inhibitor'[Title/Abstract] OR 'Checkpoint Inhibitor, Immune'[Title/Abstract] OR 'Immune Checkpoint Blockers'[Title/Abstract] OR 'Checkpoint Blockers, Immune'[Title/Abstract] OR 'Immune Checkpoint Blockade'[Title/Abstract] OR 'Checkpoint Blockade, Immune'[Title/Abstract] OR 'Immune Checkpoint Inhibition'[Title/Abstract] OR 'Checkpoint Inhibition, Immune'[Title/Abstract] OR 'PD-L1 Inhibitors'[Title/Abstract] OR 'PD L1 Inhibitors'[Title/Abstract] OR 'PD-L1 Inhibitor'[Title/Abstract] OR 'PD L1 Inhibitor'[Title/Abstract] OR 'Programmed Death-Ligand 1 Inhibitors'[Title/Abstract] OR 'Programmed Death Ligand 1 Inhibitors'[Title/Abstract] OR 'PD-1-PD-L1 Blockade'[Title/Abstract] OR 'Blockade, PD-1-PD-L1'[Title/Abstract] OR 'PD 1 PD L1 Blockade'[Title/Abstract] OR 'CTLA-4 Inhibitors'[Title/Abstract] OR 'CTLA 4 Inhibitors'[Title/Abstract] OR 'CTLA-4 Inhibitor'[Title/Abstract] OR 'CTLA 4 Inhibitor'[Title/Abstract] OR 'Cytotoxic T-Lymphocyte-Associated Protein 4 Inhibitors'[Title/Abstract] OR 'Cytotoxic T Lymphocyte Associated Protein 4 Inhibitors'[Title/Abstract] OR 'Cytotoxic T-Lymphocyte-Associated Protein 4 Inhibitor'[Title/Abstract] OR 'Cytotoxic T Lymphocyte Associated Protein 4 Inhibitor'[Title/Abstract] OR 'PD-1 Inhibitors'[Title/Abstract] OR 'PD 1 Inhibitors'[Title/Abstract] OR 'PD-1 Inhibitor'[Title/Abstract] OR 'Inhibitor, PD-1'[Title/Abstract] OR 'PD 1 Inhibitor'[Title/Abstract] OR 'Programmed Cell Death Protein 1 Inhibitor'[Title/Abstract] OR 'Programmed Cell Death Protein 1 Inhibitors'[Title/Abstract] OR pembrolizumab[Title/Abstract] OR nivolumab[Title/Abstract] OR Toripalimab[Title/Abstract] OR Sintilimab[Title/Abstract] OR Camrelizumab[Title/Abstract] OR Tislelizumab[Title/Abstract] OR Penpulimab[Title/Abstract] OR Zimberelimab[Title/Abstract] OR Serplulimab[Title/Abstract] OR Pucotenlimab[Title/Abstract] OR Atezolizumab[Title/Abstract] OR Durvalumab[Title/Abstract] OR Sugemalimab[Title/Abstract] OR Envafolimab[Title/Abstract] OR Adebrelimab[Title/Abstract] OR Ipilimumab[Title/Abstract] OR Cadonilimab[Title/Abstract]

3.1 OR 2

4."myasthenia gravis"[MeSH Terms]

5."myasthenia gravis ocular"[Title/Abstract] OR "ocular myasthenia gravis"[Title/Abstract] OR "myasthenia gravis generalized"[Title/Abstract] OR "generalized myasthenia gravis"[Title/Abstract] OR "muscle specific receptor tyrosine kinase myasthenia gravis"[Title/Abstract] OR "muscle specific receptor tyrosine kinase myasthenia gravis"[Title/Abstract] OR "muscle specific tyrosine kinase antibody positive myasthenia gravis"[Title/Abstract] OR "muscle specific tyrosine kinase antibody positive myasthenia gravis"[Title/Abstract] OR "musk mg"[Title/Abstract] OR "musk myasthenia gravis"[Title/Abstract] OR "myasthenia gravis musk"[Title/Abstract] OR "anti musk myasthenia gravis"[Title/Abstract] OR "anti musk myasthenia gravis"[Title/Abstract] OR "myasthenia gravis anti musk"[Title/Abstract]

6.4 OR 5

7.3 AND 6

二、Embase

1.immune checkpoint inhibitor'/exp

2.'checkpoint inhibitors, immune' OR 'immune checkpoint inhibitor' OR 'checkpoint inhibitor, immune' OR 'immune checkpoint blockers' OR 'checkpoint blockers, immune' OR 'immune checkpoint blockade' OR 'checkpoint blockade, immune' OR 'immune checkpoint inhibition' OR 'checkpoint inhibition, immune' OR 'pd-l1 inhibitors' OR 'pd l1 inhibitors' OR 'pd-l1 inhibitor' OR 'pd l1 inhibitor' OR 'programmed death-ligand 1 inhibitors' OR 'programmed death ligand 1 inhibitors' OR 'pd-1-pd-l1 blockade' OR 'blockade, pd-1-pd-l1' OR 'pd 1 pd l1 blockade' OR 'ctla-4 inhibitors' OR 'ctla 4 inhibitors' OR 'ctla-4 inhibitor' OR 'ctla 4 inhibitor' OR 'cytotoxic t-lymphocyte-associated protein 4 inhibitors' OR 'cytotoxic t lymphocyte associated protein 4 inhibitors' OR 'cytotoxic t-lymphocyte-associated protein 4 inhibitor' OR 'cytotoxic t lymphocyte associated protein 4 inhibitor' OR 'pd-1 inhibitors' OR 'pd 1 inhibitors' OR 'pd-1 inhibitor' OR 'inhibitor, pd-1' OR 'pd 1 inhibitor' OR 'programmed cell death protein 1 inhibitor' OR 'programmed cell death protein 1 inhibitors'

pembrolizumab OR nivolumab OR toripalimab OR sintilimab OR camrelizumab OR tislelizumab OR penpulimab OR zimberelimab OR serplulimab OR pucotenlimab OR atezolizumab OR durvalumab OR sugemalimab OR envafolimab OR adebrelimab OR ipilimumab OR cadonilimab

3.1 OR 2

4.'myasthenia gravis'/exp

5.'myasthenia gravis, ocular' OR 'ocular myasthenia gravis' OR 'myasthenia gravis, generalized' OR 'generalized myasthenia gravis' OR 'muscle-specific receptor tyrosine kinase myasthenia gravis' OR 'muscle specific receptor tyrosine kinase myasthenia gravis' OR 'muscle-specific tyrosine kinase antibody positive myasthenia gravis' OR 'muscle specific tyrosine kinase antibody positive myasthenia gravis' OR 'musk mg' OR 'musk myasthenia gravis' OR 'myasthenia gravis, musk' OR 'anti-musk myasthenia gravis' OR 'anti musk myasthenia gravis' OR 'myasthenia gravis, anti-musk'

6.4 OR 5

7.3 AND 6
