# Supplementary material for: Prognosis of immune checkpoint inhibitor-induced myasthenia gravis: a single center experience and systematic review
Source: Front Neurol. 2024 Apr 3;15:1372861. doi: 10.3389/fneur.2024.1372861 (PMC11022771; doi:10.3389/fneur.2024.1372861)
Supplement: Supplementary file 2 [file Table_2.DOCX]

Definitions for ICI-induced MG: For both PLAGH and literature identified cases, definite diagnosis of MG was considered on the basis of having ocular and/or systemic muscle weakness, and at least one of the following criteria: (1) elevated titers of anti-acetylcholine receptor (AChR) antibodies, (2) findings suggestive of MG on electrodiagnostic studies, (3) positive edrophonium test, or (4) positive ice pack test. Probable diagnosis of MG was also considered based on the neurologist’s report confirming the diagnosis of MG on the basis of high clinical suspicion alone;
