# Supplementary material for: Prognosis of immune checkpoint inhibitor-induced myasthenia gravis: a single center experience and systematic review
Source: Front Neurol. 2024 Apr 3;15:1372861. doi: 10.3389/fneur.2024.1372861 (PMC11022771; doi:10.3389/fneur.2024.1372861)
Supplement: Supplementary file 3 [file Table_3.DOCX]

**Supplementary table 3. Quality Appraisal of the Literature Reported Cases**.

| **Lead author (citation)** | **Title** | **Patient demographics** | **Current health status** | **Medical history** | **Physical exam** | **Patient disposition** | **Drug identification** | **Dosage** | **Drug reaction interface** | **Concomitant therapy** | **Adverse events** | **Discussion** |
| --- | --- | --- | --- | --- | --- | --- | --- | --- | --- | --- | --- | --- |
| [1]March et al | Yes | Yes | Yes | Yes | Yes | Yes | Yes | Yes | Yes | Yes | Yes | Yes |
| [2]Gonzalez et al | Yes | Yes | Yes | Yes | Partially | Yes | Yes | Yes | Yes | Yes | Yes | Yes |
| [3]Nguyen BH et al | Yes | Yes | Yes | Yes | Yes | Yes | Yes | Yes | Yes | Yes | Yes | Yes |
| [4]Shirai T et al | Yes | Yes | Yes | Yes | Yes | Yes | Yes | Yes | Yes | Yes | Yes | Yes |
| [5]Zimmer L et al | Yes | Yes | Yes | Yes | Yes | Yes | Yes | Yes | Yes | Yes | Yes | Yes |
| [6]Alnahhas I et al | Yes | Yes | Yes | Yes | Yes | Yes | Yes | Yes | Yes | Yes | Yes | Yes |
| [7]Polat P et al | Yes | Yes | Yes | Yes | Yes | Yes | Yes | Partially | Yes | Yes | Yes | Yes |
| [8]Sciacca G et al | Yes | Yes | Yes | Yes | Yes | Yes | Yes | Yes | Yes | Yes | Yes | Yes |
| [9]Chang E et al | Yes | Yes | Yes | Yes | Yes | Yes | Yes | Yes | Yes | Yes | Yes | Yes |
| [10]Wang C et al | Yes | Yes | Yes | Yes | Yes | Yes | Yes | Yes | Yes | Yes | Yes | Yes |
| [11]Liao B et al | Yes | Yes | Yes | Yes | Yes | Yes | Yes | Yes | Yes | Yes | Yes | Yes |
| [12]Johnson DB et al | Yes | Yes | Yes | Yes | Yes | Yes | Yes | Yes | Yes | Yes | Yes | Yes |
| [13]Montes V et al | Yes | Yes | Yes | Yes | Yes | Yes | Yes | Partially | Yes | Yes | Yes | Partially |
| [14]Pottier C et al | Yes | Yes | Yes | Yes | Yes | Yes | Yes | Yes | Yes | Yes | Yes | Yes |
| [15]Loochtan AI et al [16] | Yes | Yes | Yes | Yes | Yes | Yes | Yes | Yes | Yes | Yes | Yes | Yes |
| [16]Makarious D et al | Yes | Yes | Yes | Yes | Yes | Yes | Yes | Yes | Partially | Yes | Yes | Yes |
| [17]Lau KH et al | Yes | Yes | Yes | Yes | Yes | Yes | Yes | Yes | Yes | Yes | Yes | Partially |
| [18]Maeda O et al | Yes | Yes | Yes | Yes | Yes | Yes | Yes | Yes | Yes | Yes | Yes | Yes |
| [19]Phadke SD et al | Yes | Yes | Yes | Yes | Yes | Yes | Yes | Yes | Yes | Yes | Yes | Yes |
| [20]Zhu J et al | Yes | Yes | Yes | Yes | Yes | Yes | Yes | Yes | Yes | Yes | Yes | Yes |
| [21]Derle E et al | Yes | Yes | Yes | Yes | Yes | Yes | Yes | Yes | Yes | Yes | Yes | Yes |
| [22]Chen YH et al | Yes | Yes | Yes | Yes | Yes | Yes | Yes | Yes | Yes | Yes | Yes | Yes |
| [23]Mehta JJ et al | Yes | Yes | Yes | Yes | Yes | Yes | Yes | Partially | Yes | Yes | Yes | Yes |
| [24]Fukasawa Y et al | Yes | Yes | Yes | Yes | Yes | Yes | Yes | Partially | Yes | Yes | Yes | Yes |
| [25]Mitsune A et al | Yes | Yes | Yes | Yes | Yes | Yes | Yes | Yes | Partially | Yes | Yes | Yes |
| [26]Kang KH et al | Yes | Yes | Yes | Yes | Yes | Yes | Yes | Yes | Yes | Yes | Yes | Yes |
| [27]Earl DE et al [29] | Yes | Yes | Yes | Yes | Yes | Yes | Yes | Yes | Yes | Yes | Yes | Yes |
| [28]Hibino M et al | Yes | Yes | Yes | Yes | Yes | Yes | Yes | Yes | Yes | Yes | Yes | Partially |
| [29]Wu S et al | Yes | Yes | Yes | Yes | Yes | Yes | Yes | Yes | Yes | Yes | Yes | Yes |
| [30]Thakolwiboon S et al | Yes | Yes | Yes | Yes | Yes | Yes | Yes | Yes | Yes | Yes | Yes | Yes |
| [31]Yuen C et al | Yes | Yes | Yes | Yes | Yes | Yes | Yes | Yes | Yes | Yes | Yes | Yes |
| [32]Cham J et al | Yes | Yes | Yes | Yes | Partially | Yes | Yes | Partially | Yes | Yes | Yes | Yes |
| [33]Abidoye O et al | Yes | Yes | Yes | Yes | Yes | Yes | Yes | Yes | Yes | Yes | Yes | Yes |
| [34]Xing Q et al [36] | Yes | Yes | Yes | Yes | Yes | Yes | Yes | Yes | Yes | Yes | Yes | Yes |
| [35]Liang S et al | Yes | Yes | Yes | Yes | Yes | Yes | Yes | Yes | Yes | Yes | Yes | Partially |
| [36]Szuchan C et al | Yes | Yes | Yes | Yes | Yes | Yes | Yes | Yes | Partially | Yes | Yes | Yes |
| [37]Isami A et al | Yes | Yes | Yes | Yes | Yes | Yes | Yes | Yes | Yes | Yes | Yes | Yes |
| [38]Giglio D et al | Yes | Yes | Yes | Yes | Yes | Yes | Yes | Yes | Yes | Yes | Yes | Yes |
| [39]Gao L et al | Yes | Yes | Yes | Yes | Yes | Yes | Yes | Partially | Yes | Yes | Yes | Yes |
| [40]Sugiyama Y et al [43] | Yes | Yes | Yes | Yes | Yes | Yes | Yes | Yes | Yes | Yes | Yes | Yes |
| [41]Reyes-Bueno JA et al | Yes | Yes | Yes | Yes | Yes | Yes | Yes | Yes | Yes | Yes | Yes | Yes |
| [42]Yanase T et al | Yes | Yes | Yes | Yes | Yes | Yes | Yes | Yes | Yes | Yes | Yes | Yes |
| [43]Zhang Jet al | Yes | Yes | Yes | Yes | Yes | Yes | Yes | Yes | Partially | Yes | Yes | Yes |
| [44]Sawai T et al | Yes | Yes | Yes | Yes | Yes | Yes | Yes | Yes | Yes | Yes | Yes | Yes |
| [45]Nakanishi S et al | Yes | Yes | Yes | Yes | Yes | Yes | Yes | Yes | Yes | Yes | Yes | Yes |
| [46]Kim JS et al [50] | Yes | Yes | Yes | Yes | Yes | Yes | Yes | Yes | Yes | Yes | Yes | Yes |
| [47]Oshima Y et al | Yes | Yes | Yes | Yes | Partially | Yes | Yes | Yes | Yes | Yes | Yes | Yes |
| [48]Giovannini E et al [53] | Yes | Yes | Yes | Yes | Yes | Yes | Yes | Yes | Yes | Yes | Yes | Yes |
| [49]Lorenzo CJ et al | Yes | Yes | Yes | Yes | Yes | Yes | Yes | Yes | Yes | Yes | Yes | Yes |
| [50]Noda Tet al | Yes | Yes | Yes | Yes | Yes | Yes | Yes | Yes | Yes | Yes | Yes | Yes |
| [51]Hayakawa N et al | Yes | Yes | Yes | Yes | Partially | Yes | Yes | Yes | Yes | Yes | Yes | Yes |
| [52]Wang S et al | Yes | Yes | Yes | Yes | Yes | Yes | Yes | Yes | Yes | Yes | Yes | Yes |
| [53]Veccia A et al | Yes | Yes | Yes | Yes | Yes | Yes | Yes | Partially | Yes | Yes | Yes | Yes |
| [54]Canino F et al | Yes | Yes | Yes | Yes | Yes | Yes | Yes | Yes | Yes | Yes | Yes | Yes |
| [55]Takai M et al | Yes | Yes | Yes | Yes | Yes | Yes | Yes | Yes | Yes | Yes | Yes | Yes |
| [56]Fazel M et al | Yes | Yes | Yes | Yes | Yes | Yes | Yes | Yes | Yes | Yes | Yes | Yes |
| [57]Becquart O et al | Yes | Yes | Yes | Yes | Yes | Yes | Yes | Yes | Yes | Yes | Yes | Yes |
| [58]Chen JH et al | Yes | Yes | Yes | Yes | Yes | Yes | Yes | Partially | Yes | Yes | Yes | Yes |
| [59]Cooper DS et al | Yes | Yes | Yes | Yes | Yes | Yes | Yes | Yes | Yes | Yes | Yes | Yes |
| [60]Hasegawa Y et al | Yes | Yes | Yes | Yes | Yes | Yes | Yes | Yes | Yes | Yes | Yes | Yes |
| [61]Tan RYC et al [68] | Yes | Yes | Yes | Yes | Yes | Yes | Yes | Yes | Yes | Yes | Yes | YPartially |
| [62]Cortellini A et al | Yes | Yes | Yes | Partially | Yes | Yes | Yes | Yes | Yes | Yes | Yes | Yes |
| [63]Crusz SM et al | Yes | Yes | Yes | Yes | Yes | Yes | Yes | Yes | Yes | Yes | Yes | Yes |
| [64]Dhenin A et al | Yes | Yes | Yes | Yes | Yes | Yes | Partially | Yes | Yes | Yes | Yes | Yes |
| [65]Lara MS et al | Yes | Yes | Yes | Yes | Yes | Yes | Yes | Yes | Yes | Yes | Yes | Yes |
| [66]Liu Q et al | Yes | Yes | Yes | Yes | Yes | Yes | Yes | Yes | Yes | Yes | Yes | Yes |
| [67]So H et al | Yes | Yes | Yes | Yes | Yes | Yes | Yes | Yes | Yes | Yes | Yes | Yes |
| [68]Tedbirt B et al | Yes | Yes | Yes | Yes | Yes | Yes | Yes | Yes | Partially | Yes | Yes | Yes |
| [69]Fukazawa R et al | Yes | Yes | Yes | Yes | Yes | Yes | Yes | Yes | Yes | Yes | Yes | Yes |
| [70]Leaver PJ et al | Yes | Yes | Yes | Partially | Yes | Yes | Yes | Yes | Yes | Yes | Yes | Partially |
| [71]Montalvo Moraleda T et al | Yes | Yes | Yes | Yes | Yes | Yes | Yes | Yes | Yes | Yes | Yes | Yes |
| [72]Miñón-Fernández B et al | Yes | Yes | Yes | Yes | Yes | Yes | Yes | Yes | Yes | Yes | Yes | Yes |
| [73]Botta C et al | Yes | Yes | Yes | Yes | Yes | Yes | Yes | Yes | Yes | Yes | Yes | Partially |
| [74]Dang T et al | Yes | Yes | Yes | Yes | Yes | Yes | Yes | Yes | Yes | Yes | Yes | Yes |
| [75]Duplaine A et al | Yes | Yes | Yes | Yes | Yes | Yes | Yes | Partially | Yes | Yes | Yes | Yes |
| [76]Hajihossainlou B et al | Yes | Yes | Yes | Yes | Yes | Yes | Yes | Yes | Yes | Yes | Yes | Yes |
| [77]Heleno CT et al | Yes | Yes | Yes | Yes | Yes | Yes | Yes | Yes | Yes | Yes | Yes | Yes |
| [78]Komatsu M et al | Yes | Yes | Yes | Yes | Yes | Yes | Partially | Yes | Yes | Yes | Yes | Partially |
| [79]Ng AH et al | Yes | Yes | Yes | Yes | Yes | Yes | Yes | Yes | Yes | Yes | Yes | Yes |
| [80]Wakefield C et al | Yes | Yes | Yes | Yes | Yes | Yes | Yes | Yes | Yes | Yes | Yes | Yes |
| [81]Ziobro AS et al | Yes | Yes | Yes | Yes | Yes | Yes | Yes | Yes | Yes | Yes | Yes | Yes |
| [82]Fuentes-Antrás J et al | Yes | Yes | Yes | Yes | Yes | Yes | Yes | Yes | Yes | Yes | Yes | Yes |
| [83]García-García J et al | Yes | Yes | Yes | Yes | Yes | Yes | Yes | Yes | Yes | Yes | Yes | Yes |
| [84]Khokher W et al | Yes | Yes | Yes | Yes | Yes | Yes | Yes | Yes | Yes | Yes | Yes | Partially |
| [85]Saishu Y et al | Yes | Yes | Yes | Yes | Yes | Yes | Yes | Yes | Yes | Yes | Yes | Yes |
| [86]Soman B et al | Yes | Yes | Yes | Yes | Yes | Yes | Partially | Yes | Yes | Yes | Yes | Yes |
| [87]Kamada S et al | Yes | Yes | Yes | Yes | Yes | Yes | Yes | Yes | Yes | Yes | Yes | Yes |
| [88]Konstantina T et al | Yes | Yes | Yes | Yes | Yes | Yes | Yes | Yes | Yes | Yes | Yes | Yes |
| [89]Rota E et al | Yes | Yes | Yes | Yes | Yes | Yes | Yes | Yes | Yes | Yes | Yes | Yes |
| [90]Jeyakumar N et al | Yes | Yes | Yes | Yes | Yes | Yes | Yes | Yes | Yes | Yes | Yes | Yes |
| [91]Phua CS et al | Yes | Yes | Yes | Yes | Yes | Yes | Yes | Yes | Yes | Yes | Yes | Yes |
| [92]Portolés Hernández A et al | Yes | Yes | Yes | Yes | Yes | Yes | Partially | Yes | Yes | Yes | Yes | Yes |
| [93]Dugena O et al | Yes | Yes | Yes | Yes | Yes | Yes | Yes | Yes | Yes | Yes | Yes | Yes |
| [94]Kee W et al | Yes | Yes | Yes | Yes | Yes | Yes | Yes | Yes | Yes | Yes | Yes | Yes |
| [95]Kodama, S et al | Yes | Yes | Yes | Yes | Yes | Yes | Yes | Yes | Yes | Yes | Yes | Yes |
| [96]Kuniyoshi J et al | Yes | Yes | Yes | Yes | Yes | Yes | Yes | Yes | Yes | Yes | Yes | Yes |

To evaluate the quality of the case reports retrieved from the literature, we used the guidelines recommended by the International Society for Pharmacoepidemiology and the International Society of Pharmacovigilance for publishing adverse events reports [97]. The assessment was carried out by one investigator and cross-checked by another investigators. We only used the items reported by the guidelines as required information, without regard to the items reported as desirable or relevant. The items appraised included: i) relevance of the title to the reported information, ii) adequate description of the patient (demographics, existing health condition, relevant past medical history, physical and laboratory abnormalities, and significant morbidity or mortality), iii) adequate description of the drug (identification of generic and trade names of the drug and the manufacturer, drug dosage, duration between drug administration and adverse events, and concomitant therapy that could potentially contributes to occurrence of adverse events), iv) adequate description of the adverse events and their outcome, and v) discussion of the evidence supporting the causal association between the drug and the adverse events. Possible item ratings are yes, partially, or no. Disagreement was resolved by discussion until consensus was reached.

[1-107]

References:

1. March KL, Samarin MJ, Sodhi A, Owens RE. Pembrolizumab-induced myasthenia gravis: A fatal case report. J Oncol Pharm Pract. 2018 Mar;24(2):146-149. doi: 10.1177/1078155216687389IF: 1.3 Q4 . Epub 2017 Feb 1. PMID: 28147928IF: 1.3 Q4 .
2. Gonzalez NL, Puwanant A, Lu A, Marks SM, Živković SA. Myasthenia triggered by immune checkpoint inhibitors: New case and literature review. Neuromuscul Disord. 2017 Mar;27(3):266-268. doi: 10.1016/j.nmd.2017.01.002. Epub 2017 Jan 6. PMID: 28109638.
3. Nguyen BH, Kuo J, Budiman A, Christie H, Ali S. Two cases of clinical myasthenia gravis associated with pembrolizumab use in responding melanoma patients. Melanoma Res. 2017 Apr;27(2):152-154. doi: 10.1097/CMR.0000000000000310. PMID: 27776019.
4. Shirai T, Sano T, Kamijo F, Saito N, Miyake T, Kodaira M, Katoh N, Nishie K, Okuyama R, Uhara H. Acetylcholine receptor binding antibody-associated myasthenia gravis and rhabdomyolysis induced by nivolumab in a patient with melanoma. Jpn J Clin Oncol. 2016 Jan;46(1):86-8. doi: 10.1093/jjco/hyv158. Epub 2015 Oct 21. PMID: 26491202.
5. Zimmer L, Goldinger SM, Hofmann L, Loquai C, Ugurel S, Thomas I, Schmidgen MI, Gutzmer R, Utikal JS, Göppner D, Hassel JC, Meier F, Tietze JK, Forschner A, Weishaupt C, Leverkus M, Wahl R, Dietrich U, Garbe C, Kirchberger MC, Eigentler T, Berking C, Gesierich A, Krackhardt AM, Schadendorf D, Schuler G, Dummer R, Heinzerling LM. Neurological, respiratory, musculoskeletal, cardiac and ocular side-effects of anti-PD-1 therapy. Eur J Cancer. 2016 Jun;60:210-25. doi: 10.1016/j.ejca.2016.02.024. Epub 2016 Apr 13. PMID: 27084345.
6. Alnahhas I, Wong J. A case of new-onset antibody-positive myasthenia gravis in a patient treated with pembrolizumab for melanoma. Muscle Nerve. 2017 Jun;55(6):E25-E26. doi: 10.1002/mus.25496. Epub 2017 Mar 23. PMID: 27935080.
7. Polat P, Donofrio PD. Myasthenia gravis induced by nivolumab therapy in a patient with non-small-cell lung cancer. Muscle Nerve. 2016 Sep;54(3):507. doi: 10.1002/mus.25163. Epub 2016 May 30. PMID: 27121245.
8. Sciacca G, Nicoletti A, Rampello L, Noto L, Parra HJ, Zappia M. Benign form of myasthenia gravis after nivolumab treatment. Muscle Nerve. 2016 Sep;54(3):507-9. doi: 10.1002/mus.25212. Epub 2016 Jul 9. PMID: 27287688.
9. Chang E, Sabichi AL, Sada YH. Myasthenia Gravis After Nivolumab Therapy for Squamous Cell Carcinoma of the Bladder. J Immunother. 2017 Apr;40(3):114-116. doi: 10.1097/CJI.0000000000000161. PMID: 28234667.
10. Wang C, Zhong B, He J, Liao X. Immune checkpoint inhibitor sintilimab-induced lethal myocarditis overlapping with myasthenia gravis in thymoma patient: A case report. Medicine (Baltimore). 2023 Apr 14;102(15):e33550. doi: 10.1097/MD.0000000000033550IF: 1.6 Q3 . PMID: 37058040; PMCID: PMC10101245.
11. Liao B, Shroff S, Kamiya-Matsuoka C, Tummala S. Atypical neurological complications of ipilimumab therapy in patients with metastatic melanoma. Neuro Oncol. 2014 Apr;16(4):589-93. doi: 10.1093/neuonc/nou001. Epub 2014 Jan 30. PMID: 24482447; PMCID: PMC3956363.
12. Johnson DB, Saranga-Perry V, Lavin PJ, Burnette WB, Clark SW, Uskavitch DR, Wallace DE, Dickson MA, Kudchadkar RR, Sosman JA. Myasthenia Gravis Induced by Ipilimumab in Patients With Metastatic Melanoma. J Clin Oncol. 2015 Nov 20;33(33):e122-4. doi: 10.1200/JCO.2013.51.1683. Epub 2014 Apr 28. PMID: 24778401; PMCID: PMC4979104.
13. Montes V, Sousa S, Pita F, Guerreiro R, Carmona C. Myasthenia Gravis Induced by Ipilimumab in a Patient With Metastatic Melanoma. Front Neurol. 2018 Apr 3;9:150. doi: 10.3389/fneur.2018.00150. PMID: 29666602; PMCID: PMC5891586.
14. Pottier C, El Habnouni C, Kervarrec T, Beltran S, Samimi M. Myasthenia gravis induced by pembrolizumab in a patient with metastatic Merkel cell carcinoma. Ann Dermatol Venereol. 2022 Jun;149(2):142-145. doi: 10.1016/j.annder.2022.01.002. Epub 2022 Feb 18. PMID: 35190214.
15. Loochtan AI, Nickolich MS, Hobson-Webb LD. Myasthenia gravis associated with ipilimumab and nivolumab in the treatment of small cell lung cancer. Muscle Nerve. 2015 Aug;52(2):307-8. doi: 10.1002/mus.24648. Epub 2015 May 14. PMID: 25759003.
16. Makarious D, Horwood K, Coward JIG. Myasthenia gravis: An emerging toxicity of immune checkpoint inhibitors. Eur J Cancer. 2017 Sep;82:128-136. doi: 10.1016/j.ejca.2017.05.041. Epub 2017 Jun 27. PMID: 28666240.
17. Lau KH, Kumar A, Yang IH, Nowak RJ. Exacerbation of myasthenia gravis in a patient with melanoma treated with pembrolizumab. Muscle Nerve. 2016 Jun;54(1):157-61. doi: 10.1002/mus.25141. Epub 2016 May 19. PMID: 27065302.
18. Maeda O, Yokota K, Atsuta N, Katsuno M, Akiyama M, Ando Y. Nivolumab for the treatment of malignant melanoma in a patient with pre-existing myasthenia gravis. Nagoya J Med Sci. 2016 Feb;78(1):119-22. PMID: 27019533; PMCID: PMC4767520.
19. Phadke SD, Ghabour R, Swick BL, Swenson A, Milhem M, Zakharia Y. Pembrolizumab Therapy Triggering an Exacerbation of Preexisting Autoimmune Disease: A Report of 2 Patient Cases. J Investig Med High Impact Case Rep. 2016 Oct 25;4(4):2324709616674316. doi: 10.1177/2324709616674316IF: 1.2 . PMID: 27826593; PMCID: PMC5084516.
20. Zhu J, Li Y. Myasthenia gravis exacerbation associated with pembrolizumab. Muscle Nerve. 2016 Sep;54(3):506-7. doi: 10.1002/mus.25055. Epub 2016 Mar 12. PMID: 26802533.
21. Derle E, Benli S. Ipilimumab treatment associated with myasthenic crises and unfavorable disease course. Neurol Sci. 2018 Oct;39(10):1773-1774. doi: 10.1007/s10072-018-3471-6. Epub 2018 Jun 13. PMID: 29948466.
22. Chen YH, Liu FC, Hsu CH, Chian CF. Nivolumab-induced myasthenia gravis in a patient with squamous cell lung carcinoma: Case report. Medicine (Baltimore). 2017 Jul;96(27):e7350. doi: 10.1097/MD.0000000000007350. PMID: 28682883; PMCID: PMC5502156.
23. Mehta JJ, Maloney E, Srinivasan S, Seitz P, Cannon M. Myasthenia Gravis Induced by Nivolumab: A Case Report. Cureus. 2017 Sep 20;9(9):e1702. doi: 10.7759/cureus.1702. PMID: 29159009; PMCID: PMC5690486.
24. Fukasawa Y, Sasaki K, Natsume M, Nakashima M, Ota S, Watanabe K, Takahashi Y, Kondo F, Kozuma K, Seki N. Nivolumab-Induced Myocarditis Concomitant with Myasthenia Gravis. Case Rep Oncol. 2017 Sep 6;10(3):809-812. doi: 10.1159/000479958. PMID: 29070994; PMCID: PMC5649238.
25. Mitsune A, Yanagisawa S, Fukuhara T, Miyauchi E, Morita M, Ono M, Tojo Y, Ichinose M. Relapsed Myasthenia Gravis after Nivolumab Treatment. Intern Med. 2018 Jul 1;57(13):1893-1897. doi: 10.2169/internalmedicine.9153-17. Epub 2018 Feb 9. PMID: 29434145; PMCID: PMC6064691.
26. Kang KH, Grubb W, Sawlani K, Gibson MK, Hoimes CJ, Rogers LR, Lavertu P, Yao M. Immune checkpoint-mediated myositis and myasthenia gravis: A case report and review of evaluation and management. Am J Otolaryngol. 2018 Sep-Oct;39(5):642-645. doi: 10.1016/j.amjoto.2018.06.003. Epub 2018 Jun 5. PMID: 29903623.
27. Earl DE, Loochtan AI, Bedlack RS. Refractory myasthenia gravis exacerbation triggered By pembrolizumab. Muscle Nerve. 2018 Apr;57(4):E120-E121. doi: 10.1002/mus.26021. Epub 2017 Dec 7. PMID: 29193151.
28. Hibino M, Maeda K, Horiuchi S, Fukuda M, Kondo T. Pembrolizumab-induced myasthenia gravis with myositis in a patient with lung cancer. Respirol Case Rep. 2018 Aug 7;6(7):e00355. doi: 10.1002/rcr2.355. PMID: 30094028; PMCID: PMC6079932.
29. Wu S, Shi J, Guan Y, Zhang L, Wang H. Successful Management of Generalized Myasthenia Gravis Induced by Atezolizumab in a Patient With Extensive-Stage SCLC: A Case Report. JTO Clin Res Rep. 2022 Jun 2;3(11):100354. doi: 10.1016/j.jtocrr.2022.100354. PMID: 36246044; PMCID: PMC9563168.
30. Thakolwiboon S, Karukote A, Wilms H. De Novo Myasthenia Gravis Induced by Atezolizumab in a Patient with Urothelial Carcinoma. Cureus. 2019 Jun 25;11(6):e5002. doi: 10.7759/cureus.5002. PMID: 31497433; PMCID: PMC6713254.
31. Yuen C, Fleming G, Meyers M, Soliven B, Rezania K. Myasthenia gravis induced by avelumab. Immunotherapy. 2019 Oct;11(14):1181-1185. doi: 10.2217/imt-2019-0106. Epub 2019 Aug 29. PMID: 31462152.
32. Cham J, Ng D, Nicholson L. Durvalumab-induced myocarditis, myositis, and myasthenia gravis: a case report. J Med Case Rep. 2021 May 31;15(1):278. doi: 10.1186/s13256-021-02858-7. PMID: 34053457; PMCID: PMC8165972.
33. Abidoye O, Kim N, Fombi J. An Interesting Case Report of Myasthenia Gravis Exacerbation Induced by Durvalumab. Cureus. 2022 Jul 18;14(7):e26985. doi: 10.7759/cureus.26985. PMID: 35989744; PMCID: PMC9381854.
34. Xing Q, Zhang ZW, Lin QH, Shen LH, Wang PM, Zhang S, Fan M, Zhu B. Myositis-myasthenia gravis overlap syndrome complicated with myasthenia crisis and myocarditis associated with anti-programmed cell death-1 (sintilimab) therapy for lung adenocarcinoma. Ann Transl Med. 2020 Mar;8(5):250. doi: 10.21037/atm.2020.01.79. PMID: 32309397; PMCID: PMC7154453.
35. Liang S, Yang J, Lin Y, Li T, Zhao W, Zhao J, Dong C. Immune Myocarditis Overlapping With Myasthenia Gravis Due to Anti-PD-1 Treatment for a Chordoma Patient: A Case Report and Literature Review. Front Immunol. 2021 Jul 8;12:682262. doi: 10.3389/fimmu.2021.682262IF: 7.3 Q1 . PMID: 34305915; PMCID: PMC8297709.
36. Szuchan C, Elson L, Alley E, Leung K, Camargo AL, Elimimian E, Nahleh Z, Sadler D. Checkpoint inhibitor-induced myocarditis and myasthenia gravis in a recurrent/metastatic thymic carcinoma patient: a case report. Eur Heart J Case Rep. 2020 Apr 7;4(3):1-8. doi: 10.1093/ehjcr/ytaa051. PMID: 32617460; PMCID: PMC7319805.
37. Isami A, Uchiyama A, Shimaoka Y, Suzuki S, Kawachi I, Fujita N. [A case of anti-titin antibody positive nivolumab-related necrotizing myopathy with myasthenia gravis]. Rinsho Shinkeigaku. 2019 Jul 31;59(7):431-435. Japanese. doi: 10.5692/clinicalneurol.cn-001270. Epub 2019 Jun 27. PMID: 31243249.
38. Giglio D, Berntsson H, Fred Å, Ny L. Immune Checkpoint Inhibitor-Induced Polymyositis and Myasthenia Gravis with Fatal Outcome. Case Rep Oncol. 2020 Oct 14;13(3):1252-1257. doi: 10.1159/000510740. PMID: 33250739; PMCID: PMC7670383.
39. Gao L, Li X, Guo Z, Tang L, Peng J, Liu B. Immune checkpoint inhibitor-induced myocarditis with myasthenia gravis overlap syndrome: A case report and literature review. Medicine (Baltimore). 2022 Dec 9;101(49):e32240. doi: 10.1097/MD.0000000000032240. PMID: 36626474; PMCID: PMC9750698.
40. Sugiyama Y, Esa Y, Watanabe A, Kobayashi J, Suzuki S, Takahashi D. [Immune checkpoint inhibitor-induced anti-striational antibodies in myasthenia gravis and myositis: a case report]. Rinsho Shinkeigaku. 2021 Sep 28;61(9):630-634. Japanese. doi: 10.5692/clinicalneurol.cn-001604. Epub 2021 Aug 26. PMID: 34433744.
41. Reyes-Bueno JA, Rodriguez-Santos L, Serrano-Castro PJ. Miastenia grave inducida por tratamiento con inhibidores del punto de control inmunologico: primer caso secundario a avelumab y revision de casos previamente publicados [Myasthenia gravis induced by inmuno checkpoints inhibitors: first case report secondary to avelumab therapy and review of published cases]. Rev Neurol. 2019 Apr 16;68(8):333-338. Spanish. doi: 10.33588/rn.6808.2018497. PMID: 30963530.
42. Yanase T, Moritoki Y, Kondo H, Ueyama D, Akita H, Yasui T. Myocarditis and myasthenia gravis by combined nivolumab and ipilimumab immunotherapy for renal cell carcinoma: A case report of successful management. Urol Case Rep. 2020 Nov 28;34:101508. doi: 10.1016/j.eucr.2020.101508. PMID: 33318935; PMCID: PMC7726655.
43. Zhang J, Li J, Zhai L, Lin L. Coexisting of myasthenia gravis and fulminant myocarditis induced by nivolumab in a patient with ureteral epithelial cancer. Neuro Endocrinol Lett. 2021 Oct;42(6):383-386. PMID: 34713689.
44. Sawai T, Hosokawa T, Shigekiyo T, Ogawa S, Sano E, Arawaka S. [An autopsy case of nivolumab-induced myasthenia gravis and myositis]. Rinsho Shinkeigaku. 2019 Jun 22;59(6):360-364. Japanese. doi: 10.5692/clinicalneurol.cn-001282. Epub 2019 May 29. PMID: 31142711.
45. Nakanishi S, Nishida S, Miyazato M, Goya M, Saito S. A case report of nivolumab-induced myasthenia gravis and myositis in a metastatic renal cell carcinoma patient. Urol Case Rep. 2019 Dec 14;29:101105. doi: 10.1016/j.eucr.2019.101105. PMID: 31908963; PMCID: PMC6940690.
46. Kim JS, Nam TS, Kim J, Kho BG, Park CK, Oh IJ, Kim YC. Myasthenia gravis and myopathy after nivolumab treatment for non-small cell lung carcinoma: A case report. Thorac Cancer. 2019 Oct;10(10):2045-2049. doi: 10.1111/1759-7714.13177. Epub 2019 Aug 21. PMID: 31436031; PMCID: PMC6775222.
47. Oshima Y, Fujii S, Horiuchi K. Pembrolizumab-induced Myasthenia Gravis Relapse after Immunosuppressive Therapy. Intern Med. 2022 Nov 1;61(21):3281-3285. doi: 10.2169/internalmedicine.8554-21. Epub 2022 Apr 9. PMID: 35400705; PMCID: PMC9683818.
48. Giovannini E, Bonasoni MP, D'Aleo M, Tamagnini I, Tudini M, Fais P, Pelotti S. Pembrolizumab-Induced Fatal Myasthenia, Myocarditis, and Myositis in a Patient with Metastatic Melanoma: Autopsy, Histological, and Immunohistochemical Findings-A Case Report and Literature Review. Int J Mol Sci. 2023 Jun 30;24(13):10919. doi: 10.3390/ijms241310919. PMID: 37446095; PMCID: PMC10342066.
49. Lorenzo CJ, Fitzpatrick H, Campdesuner V, George J, Lattanzio N. Pembrolizumab-Induced Ocular Myasthenic Crisis. Cureus. 2020 Jul 14;12(7):e9192. doi: 10.7759/cureus.9192. PMID: 32685327; PMCID: PMC7366038.
50. Noda T, Kageyama H, Miura M, Tamura T, Ito H. [A case of myasthenia gravis and myositis induced by pembrolizumab]. Rinsho Shinkeigaku. 2019 Aug 29;59(8):502-508. Japanese. doi: 10.5692/clinicalneurol.cn-001251. Epub 2019 Jul 23. PMID: 31341124.
51. Hayakawa N, Kikuchi E, Suzuki S, Oya M. Myasthenia gravis with myositis induced by pembrolizumab therapy in a patient with metastatic urothelial carcinoma. Int Cancer Conf J. 2020 Mar 21;9(3):123-126. doi: 10.1007/s13691-020-00408-4. PMID: 32582515; PMCID: PMC7297883.
52. Wang S, Peng D, Zhu H, Min W, Xue M, Wu R, Shao Y, Pan L, Zhu M. Acetylcholine receptor binding antibody-associated myasthenia gravis, myocarditis, and rhabdomyolysis induced by tislelizumab in a patient with colon cancer: A case report and literature review. Front Oncol. 2022 Dec 8;12:1053370. doi: 10.3389/fonc.2022.1053370. PMID: 36568231; PMCID: PMC9773380.
53. Veccia A, Kinspergher S, Grego E, Peterlana D, Berti A, Tranquillini E, Caffo O. Myositis and myasthenia during nivolumab administration for advanced lung cancer: a case report and review of the literature. Anticancer Drugs. 2020 Jun;31(5):540-544. doi: 10.1097/CAD.0000000000000903. PMID: 32011360.
54. Canino F, Pugliese G, Baldessari C, Greco S, Depenni R, Dominici M. Cemiplimab- and nivolumab-induced myasthenia gravis: two clinical cases. Tumori. 2021 Dec;107(6):NP123-NP126. doi: 10.1177/03008916211040559. Epub 2021 Aug 21. PMID: 34423700.
55. Takai M, Kato D, Iinuma K, Maekawa YM, Nakane K, Tsuchiya T, Yokoi S, Koie T. Simultaneous pembrolizumab-induced myasthenia gravis and myocarditis in a patient with metastatic bladder cancer: A case report. Urol Case Rep. 2020 Feb 26;31:101145. doi: 10.1016/j.eucr.2020.101145. PMID: 32190548; PMCID: PMC7068635.
56. Fazel M, Jedlowski PM. Severe Myositis, Myocarditis, and Myasthenia Gravis with Elevated Anti-Striated Muscle Antibody following Single Dose of Ipilimumab-Nivolumab Therapy in a Patient with Metastatic Melanoma. Case Reports Immunol. 2019 Apr 30;2019:2539493. doi: 10.1155/2019/2539493IF: 1.0 . PMID: 31183226IF: 1.0 ; PMCID: PMC6515062IF: 1.0 .
57. Becquart O, Lacotte J, Malissart P, Nadal J, Lesage C, Guillot B, Du Thanh A. Myasthenia Gravis Induced by Immune Checkpoint Inhibitors. J Immunother. 2019 Oct;42(8):309-312. doi: 10.1097/CJI.0000000000000278. PMID: 31246640.
58. Chen JH, Lee KY, Hu CJ, Chung CC. Coexisting myasthenia gravis, myositis, and polyneuropathy induced by ipilimumab and nivolumab in a patient with non-small-cell lung cancer: A case report and literature review. Medicine (Baltimore). 2017 Dec;96(50):e9262. doi: 10.1097/MD.0000000000009262IF: 1.6 Q3 . PMID: 29390370; PMCID: PMC5815782.
59. Cooper DS, Meriggioli MN, Bonomi PD, Malik R. Severe Exacerbation of Myasthenia Gravis Associated with Checkpoint Inhibitor Immunotherapy. J Neuromuscul Dis. 2017;4(2):169-173. doi: 10.3233/JND-170219. PMID: 28505981.
60. Hasegawa Y, Kawai S, Ota T, Tsukuda H, Fukuoka M. Myasthenia gravis induced by nivolumab in patients with non-small-cell lung cancer: a case report and literature review. Immunotherapy. 2017 Aug;9(9):701-707. doi: 10.2217/imt-2017-0043IF: 2.8 Q4 . Epub 2017 Jun 26. PMID: 28649876.
61. Tan RYC, Toh CK, Takano A. Continued Response to One Dose of Nivolumab Complicated by Myasthenic Crisis and Myositis. J Thorac Oncol. 2017 Jul;12(7):e90-e91. doi: 10.1016/j.jtho.2017.02.024IF: 20.4 Q1 . PMID: 28629544IF: 20.4 Q1 .
62. Cortellini A, Napoleoni L, Cimini N, Parisi A, Pavese F, DʼOrazio C, Verna L, Porzio G, Ficorella C. Immune Checkpoint Inhibitors and Myasthenic Syndromes: A Case Report of a Metastatic Renal Cell Carcinoma Patient Treated With Nivolumab. J Clin Neuromuscul Dis. 2018 Dec;20(2):99-100. doi: 10.1097/CND.0000000000000220. PMID: 30439756.
63. Crusz SM, Radunovic A, Shepherd S, Shah S, Newey V, Phillips M, Lim L, Powles T, Szlosarek PW, Shamash J, Rashid S. Rituximab in the treatment of pembrolizumab-induced myasthenia gravis. Eur J Cancer. 2018 Oct;102:49-51. doi: 10.1016/j.ejca.2018.07.125IF: 8.4 Q1 . Epub 2018 Aug 20. PMID: 30138772IF: 8.4 Q1 .
64. Dhenin A, Samartzi V, Lejeune S, Seront E. Cascade of immunologic adverse events related to pembrolizumab treatment. BMJ Case Rep. 2019 Jun 4;12(6):e229149. doi: 10.1136/bcr-2018-229149IF: 0.9 . PMID: 31167767; PMCID: PMC6557364.
65. Lara MS, Afify A, Ellis MP, Phan CT, Richman DP, Riess JW. Immune Checkpoint Inhibitor-Induced Myasthenia Gravis in a Patient with Advanced NSCLC and Remote History of Thymoma. Clin Lung Cancer. 2019 Jul;20(4):e489-e491. doi: 10.1016/j.cllc.2019.04.007IF: 3.6 Q2 . Epub 2019 Apr 19. PMID: 31085042.
66. Liu Q, Ayyappan S, Broad A, Narita A. Pembrolizumab-associated ocular myasthenia gravis. Clin Exp Ophthalmol. 2019 Aug;47(6):796-798. doi: 10.1111/ceo.13499IF: 4.0 Q1 . Epub 2019 Apr 1. PMID: 30859689IF: 4.0 Q1 .
67. So H, Ikeguchi R, Kobayashi M, Suzuki M, Shimizu Y, Kitagawa K. PD-1 inhibitor-associated severe myasthenia gravis with necrotizing myopathy and myocarditis. J Neurol Sci. 2019 Apr 15;399:97-100. doi: 10.1016/j.jns.2019.02.023IF: 4.4 Q2 . Epub 2019 Feb 13. PMID: 30798111.
68. Tedbirt B, De Pontville M, Branger P, Picard C, Baroudjian B, Lebbé C, Carpentier AF, Delyon J; PATIO group. Rechallenge of immune checkpoint inhibitor after pembrolizumab-induced myasthenia gravis. Eur J Cancer. 2019 May;113:72-74. doi: 10.1016/j.ejca.2019.03.006IF: 8.4 Q1 . Epub 2019 Apr 12. PMID: 30986705.
69. Fukazawa R, Takezawa H, Tsuji Y, Noto Y, Banba M, Fujii A. [A case of myasthenia gravis developed during pembrolizumab administration, suggesting an excitation-contraction connection disorder]. Rinsho Shinkeigaku. 2020 Jan 30;60(1):37-40. Japanese. doi: 10.5692/clinicalneurol.cn-001323. Epub 2019 Dec 17. PMID: 31852868.
70. Leaver PJ, Jang HS, Vernon ST, Fernando SL. Immune checkpoint inhibitor-mediated myasthenia gravis with focal subclinical myocarditis progressing to symptomatic cardiac disease. BMJ Case Rep. 2020 May 13;13(5):e232920. doi: 10.1136/bcr-2019-232920IF: 0.9 . PMID: 32404371; PMCID: PMC7228148.
71. Montalvo Moraleda T, Horga A, Galán Dávila L, Guerrero Sola A, Silva Hernández L. Nivolumab: An «Immune storm» in a patient with history of myasthenia gravis. Neurologia (Engl Ed). 2020 Nov-Dec;35(9):692-694. English, Spanish. doi: 10.1016/j.nrl.2019.11.007. Epub 2020 Jan 28. PMID: 32005531.
72. Miñón-Fernández B, Losada-Domingo JM, Sánchez-Horvath MT, Bárcena-Llona J. Miastenia grave asociada a nivolumab [Myasthenia gravis associated with nivolumab]. Rev Neurol. 2020 Jan 16;70(2):72-73. Spanish. doi: 10.33588/rn.7002.2019153IF: 1.2 Q4 . PMID: 31930473IF: 1.2 Q4 .
73. Botta C, Agostino RM, Dattola V, Cianci V, Calandruccio ND, Bianco G, Mafodda A, Maisano R, Iuliano E, Orizzonte G, Mazzacuva D, Falzea AC, Saladino RE, Giannicola R, Restifo G, Aguglia U, Caraglia M, Correale P. Myositis/Myasthenia after Pembrolizumab in a Bladder Cancer Patient with an Autoimmunity-Associated HLA: Immune-Biological Evaluation and Case Report. Int J Mol Sci. 2021 Jun 10;22(12):6246. doi: 10.3390/ijms22126246IF: 5.6 Q1 . PMID: 34200673; PMCID: PMC8230397.
74. Dang T, Macwan S, Dasanu CA. Late-onset double-seronegative myasthenia gravis syndrome and myasthenic crisis due to nivolumab use for Hodgkin's lymphoma. J Oncol Pharm Pract. 2021 Sep;27(6):1534-1538. doi: 10.1177/1078155220976797IF: 1.3 Q4 . Epub 2020 Dec 8. PMID: 33292071IF: 1.3 Q4 .
75. Duplaine A, Prot C, Le-Masson G, Soulages A, Duval F, Dutriaux C, Prey S. Myasthenia Gravis Lambert-Eaton overlap syndrome induced by nivolumab in a metastatic melanoma patient. Neurol Sci. 2021 Dec;42(12):5377-5378. doi: 10.1007/s10072-021-05557-9. Epub 2021 Aug 24. PMID: 34427792.
76. Hajihossainlou B, Vasileva A, Manthri S, Chakraborty K. Myasthenia gravis induced or exacerbated by immune checkpoint inhibitors: a rising concern. BMJ Case Rep. 2021 Aug 23;14(8):e243764. doi: 10.1136/bcr-2021-243764IF: 0.9 . PMID: 34426425; PMCID: PMC8383870.
77. Heleno CT, Mustafa A, Gotera NA, Tesar A. Myasthenia Gravis as an Immune-Mediated Side Effect of Checkpoint Inhibitors. Cureus. 2021 Jul 11;13(7):e16316. doi: 10.7759/cureus.16316IF: 1.2 . PMID: 34277306IF: 1.2 ; PMCID: PMC8275066.
78. Komatsu M, Hirai M, Kobayashi K, Hashidate H, Fukumoto J, Sato A, Usuda H, Tanaka K, Takahashi K, Kuwabara S. A rare case of nivolumab-related myasthenia gravis and myocarditis in a patient with metastatic gastric cancer. BMC Gastroenterol. 2021 Aug 26;21(1):333. doi: 10.1186/s12876-021-01904-4IF: 2.4 Q4 . PMID: 34445963; PMCID: PMC8393464.
79. Ng AH, Molinares DM, Ngo-Huang AT, Bruera E. Immunotherapy-related skeletal muscle weakness in cancer patients: a case series. Ann Palliat Med. 2021 Feb;10(2):2359-2365. doi: 10.21037/apm-20-454. Epub 2020 Jun 21. PMID: 32575996.
80. Wakefield C, Shultz C, Patel B, Malla M. Life-threatening immune checkpoint inhibitor-induced myocarditis and myasthenia gravis overlap syndrome treated with abatacept: a case report. BMJ Case Rep. 2021 Nov 2;14(11):e244334. doi: 10.1136/bcr-2021-244334IF: 0.9 . PMID: 34728505; PMCID: PMC8565525.
81. Ziobro AS, LaPlante RL, DeMari SR, Clark LM, Kingsley DJ, Smith AJ. Myasthenia Gravis Associated With Programmed Death-1 (PD-1) Receptor Inhibitor Pembrolizumab: A 40-day Case Report. J Pharm Pract. 2021 Feb;34(1):166-170. doi: 10.1177/0897190020970750IF: 1.3 . Epub 2020 Nov 4. PMID: 33143531.
82. Fuentes-Antrás J, Peinado P, Guevara-Hoyer K, Díaz Del Arco C, Sánchez-Ramón S, Aguado C. Fatal Autoimmune Storm After a Single Cycle of Anti-PD-1 Therapy: A Case of Lethal Toxicity but Pathological Complete Response in Metastatic Lung Adenocarcinoma. Hematol Oncol Stem Cell Ther. 2022 Mar 1;15(1):63-67. doi: 10.1016/j.hemonc.2020.04.006. PMID: 32442551.
83. García-García J, Díaz-Maroto I, González-Villar E, Martínez-Martín A. Miastenia gravis inducida por atezolizumab [Myasthenia gravis induced by atezolizumab]. Rev Neurol. 2022 May 16;74(10):343-344. Spanish. doi: 10.33588/rn.7410.2021264IF: 1.2 Q4 . PMID: 35548916.
84. Khokher W, Bhuta S, Kesireddy N, Abuhelwa Z, Mhanna M, Iftikhar S, Horen N, Assaly R. Refractory Nivolumab-Induced Myasthenia Gravis Treated With Abatacept. Am J Ther. 2022 Sep-Oct 01;29(5):e576-e577. doi: 10.1097/MJT.0000000000001405IF: 4.2 Q2 . Epub 2021 Jul 9. PMID: 34264884.
85. Saishu Y, Yoshida T, Seino Y, Nomura T. Nivolumab-related myasthenia gravis with myositis requiring prolonged mechanical ventilation: a case report. J Med Case Rep. 2022 Feb 14;16(1):61. doi: 10.1186/s13256-022-03286-xIF: 1.0 . PMID: 35152911IF: 1.0 ; PMCID: PMC8842808IF: 1.0 .
86. Soman B, Dias MC, Rizvi SAJ, Kardos A. Myasthenia gravis, myositis and myocarditis: a fatal triad of immune-related adverse effect of immune checkpoint inhibitor treatment. BMJ Case Rep. 2022 Dec 8;15(12):e251966. doi: 10.1136/bcr-2022-251966IF: 0.9 . PMID: 36593626; PMCID: PMC9743272.
87. Kamada S, Hanazono A, Sanpei Y, et al. Alternative to steroid therapy for myasthenia gravis and myositis occurring as immune‐related adverse events[J]. Clinical and Experimental Neuroimmunology, 2019, 10(3): 190-191.
88. Konstantina T, Konstantinos R, Anastasios K, et al. Fatal adverse events in two thymoma patients treated with anti-PD-1 immune check point inhibitor and literature review[J]. Lung cancer, 2019, 135: 29-32.
89. Rota E, Varese P, Agosti S, Celli L, Ghiglione E, Pappalardo I, Zaccone G, Paglia A, Morelli N. Concomitant myasthenia gravis, myositis, myocarditis and polyneuropathy, induced by immune-checkpoint inhibitors: A life-threatening continuum of neuromuscular and cardiac toxicity. eNeurologicalSci. 2018 Nov 22;14:4-5. doi: 10.1016/j.ensci.2018.11.023. PMID: 30533536; PMCID: PMC6262799.
90. Jeyakumar N, Etchegaray M, Henry J, Lelenwa L, Zhao B, Segura A, Buja LM. The Terrible Triad of Checkpoint Inhibition: A Case Report of Myasthenia Gravis, Myocarditis, and Myositis Induced by Cemiplimab in a Patient with Metastatic Cutaneous Squamous Cell Carcinoma. Case Reports Immunol. 2020 Jul 4;2020:5126717. doi: 10.1155/2020/5126717IF: 1.0 . PMID: 32695533IF: 1.0 ; PMCID: PMC7355354IF: 1.0 .
91. Phua CS, Murad A, Fraser C, Bray V, Cappelen-Smith C. Myasthenia gravis and concurrent myositis following PD-L1 checkpoint inhibitor for non-small cell lung cancer. BMJ Neurol Open. 2020 Mar 19;2(1):e000028. doi: 10.1136/bmjno-2019-000028IF: 2.7 . PMID: 33681778; PMCID: PMC7871726.
92. Portolés Hernández A, Blanco Clemente M, Escribano García D, Velasco Calvo R, Núñez García B, Oteo Domínguez JF, Salas Antón C, Méndez García M, Segovia Cubero J, Domínguez F. Checkpoint inhibitor-induced fulminant myocarditis, complete atrioventricular block and myasthenia gravis-a case report. Cardiovasc Diagn Ther. 2021 Aug;11(4):1013-1019. doi: 10.21037/cdt-21-147IF: 2.4 Q3 . PMID: 34527524IF: 2.4 Q3 ; PMCID: PMC8410500IF: 2.4 Q3 .
93. Dugena O, Zheng C, Taylor J, Wong A. Pembrolizumab-induced Myasthenia Gravis: Literature Review of Ocular Manifestations and a Refractory Case. J Immunother. 2022 Jul-Aug 01;45(6):267-273. doi: 10.1097/CJI.0000000000000422IF: 3.9 Q2 . Epub 2022 May 17. PMID: 35575994.
94. Kee W, Ng KYY, Lee JJX, Tan DSW. Myasthenia Gravis and Myocarditis After Administration of Pembrolizumab in a Patient With Metastatic Non-small Cell Lung Cancer and Resected Thymoma. Clin Lung Cancer. 2022 Jun;23(4):e293-e295. doi: 10.1016/j.cllc.2021.12.001IF: 3.6 Q2 . Epub 2021 Dec 10. PMID: 35105525IF: 3.6 Q2 .
95. Kodama, S.Yoshida, M.Miki, H.Ito, T.Goto, H.Terashima, T.Fujiwara, A.Hayashi, K.Kamon, T.A Case of Myocarditis-combined Myasthenia Gravis after Combination Therapy with Ipilimumab and Nivolumab for Pleomorphic Carcinoma of the LungJapanese Journal of Lung Cancer 2022 Vol. 62 Issue 1 Pages 57-64DOI: 10.2482/haigan.62.57
96. Kuniyoshi J, Huang R, Choi H, Bernas M, Techasatian W, Nishimura Y. Immune Checkpoint Inhibitor-Induced Myocarditis With Concurrent Myasthenia Gravis. Can J Cardiol. 2023 Jun 15:S0828-282X(23)00516-0. doi: 10.1016/j.cjca.2023.06.007IF: 6.2 Q1 . Epub ahead of print. PMID: 37330137IF: 6.2 Q1 .
97. Kelly WN, Arellano FM, Barnes J, Bergman U, Edwards IR, Fernandez AM, et al. Guidelines for submitting adverse event reports for publication. Pharmacoepidemiology and drug safety. 2007;16(5):581-7. Epub 2007/05/02. doi: 10.1002/pds.1399. PubMed PMID: 17471601.
