# Supplementary material for: Prognosis of immune checkpoint inhibitor-induced myasthenia gravis: a single center experience and systematic review
Source: Front Neurol. 2024 Apr 3;15:1372861. doi: 10.3389/fneur.2024.1372861 (PMC11022771; doi:10.3389/fneur.2024.1372861)
Supplement: Supplementary file 4 [file Table_4.DOCX]

Supplementary Table 4. Demographic, oncological characteristics, in the 110 patients included in the cohort with neurological toxicities from ICI treatment.

| Patient ID | Age | Gender | Patient's Origin | Type of Cancer | ICI | Cycles completed | Onset (weeks) |
| --- | --- | --- | --- | --- | --- | --- | --- |
|  |  | 1 Male, 2 Female |  | 1=Melanoma；2=Lung Carcinoma；3=renal Carcinoma；4=others | 1=PD(L)-1；2=CTLA-4；3=PD-1+CTLA-4 | 1=1  2=2  3=≥3 | 1=≤4  2=>4 |
|  | 79 | 1 | PLAGH | 4 | 1 | 3 | 2 |
|  | 67 | 1 | PLAGH | 2 | 1 | 3 | 2 |
|  | 62 | 2 | PLAGH | 4 | 1 | 2 | 1 |
|  | 76 | 1 | PLAGH | 2 | 1 | 1 | 1 |
|  | 66 | 1 | PLAGH | 4 | 1 | 2 | 1 |
|  | 64 | 2 | PLAGH | 4 | 1 | 1 | 1 |
|  | 75 | 2 | PLAGH | 2 | 1 | 1 | 1 |
|  | 49 | 1 | PLAGH | 4 | 1 | 3 | 1 |
|  | 58 | 1 | PLAGH | 2 | 1 | 3 | 2 |
|  | 71 | 2 | Reported Cases | 4 | 1 | 3 | 2 |
|  | 81 | 1 | Reported Cases | 1 | 1 | 3 | 2 |
|  | 86 | 2 | Reported Cases | 1 | 1 | 2 | 2 |
|  | 81 | 2 | Reported Cases | 1 | 1 | 1 | 1 |
|  | 69 | 2 | Reported Cases | 1 | 1 | 3 | 2 |
|  | 84 | 1 | Reported Cases | 1 | 1 | 2 | 2 |
|  | 65 | 1 | Reported Cases | 2 | 1 | 3 | 2 |
|  | 81 | 1 | Reported Cases | 2 | 1 | 3 | 2 |
|  | 75 | 1 | Reported Cases | 4 | 1 | 2 | 2 |
|  | 74 | 2 | Reported Cases | 4 | 1 | 2 | 2 |
|  | 75 | 1 | Reported Cases | 1 | 1 | 1 | 2 |
|  | 79 | 1 | Reported Cases | 1 | 1 | 3 | 2 |
|  | 75 | 1 | Reported Cases | 1 | 1 | 2 | 2 |
|  | 59 | 2 | Reported Cases | 1 | 1 | 3 | 2 |
|  | 85 | 2 | Reported Cases | 1 | 1 | 2 | 1 |
|  | 73 | 1 | Reported Cases | 3 | 1 | 2 | 1 |
|  | 74 | 1 | Reported Cases | 1 | 1 | 2 | 2 |
|  | 56 | 1 | Reported Cases | 2 | 1 | 3 | 2 |
|  | 87 | 1 | Reported Cases | 4 | 1 | 2 | 1 |
|  | 74 | 2 | Reported Cases | 4 | 1 | 3 | 2 |
|  | 82 | 2 | Reported Cases | 2 | 1 | 3 | 2 |
|  | 53 | 1 | Reported Cases | 2 | 1 | 1 | 1 |
|  | 65 | 1 | Reported Cases | 2 | 1 | 1 | 2 |
|  | 76 | 1 | Reported Cases | 2 | 1 | 3 | 2 |
|  | 74 | 1 | Reported Cases | 4 | 1 | 3 | 2 |
|  | 67 | 1 | Reported Cases | 4 | 1 | 2 | 2 |
|  | 90 | 1 | Reported Cases | 2 | 1 | 2 | 2 |
|  | 75 | 1 | Reported Cases | 1 | 1 | 1 | 1 |
|  | 75 | 2 | Reported Cases | 1 | 1 | 3 | 2 |
|  | 68 | 2 | Reported Cases | 2 | 1 | 3 | 2 |
|  | 76 | 2 | Reported Cases | 2 | 1 | 2 | 1 |
|  | 78 | 2 | Reported Cases | 3 | 1 | 3 | 2 |
|  | 79 | 2 | Reported Cases | 2 | 1 | 3 | 2 |
|  | 63 | 2 | Reported Cases | 2 | 1 | 2 | 1 |
|  | 73 | 1 | Reported Cases | 1 | 1 | 2 | 2 |
|  | 77 | 1 | Reported Cases | 1 | 1 | 3 | 2 |
|  | 50 | 2 | Reported Cases | 4 | 1 | 2 | 1 |
|  | 80 | 1 | Reported Cases | 1 | 1 | 3 | 2 |
|  | 72 | 1 | Reported Cases | 4 | 1 | 3 | 2 |
|  | 34 | 2 | Reported Cases | 4 | 1 | 3 | 2 |
|  | 78 | 1 | Reported Cases | 1 | 1 | 3 | 2 |
|  | 66 | 1 | Reported Cases | 1 | 1 | 2 | 1 |
|  | 43 | 2 | Reported Cases | 4 | 1 | 1 | 2 |
|  | 65 | 1 | Reported Cases | 3 | 1 | 3 | 2 |
|  | 76 | 1 | Reported Cases | 1 | 1 | 2 | 1 |
|  | 72 | 1 | Reported Cases | 4 | 1 | 2 | 1 |
|  | 77 | 2 | Reported Cases | 1 | 1 | 2 | 2 |
|  | 69 | 2 | Reported Cases | 1 | 2 | 3 | 2 |
|  | 73 | 2 | Reported Cases | 1 | 2 | 2 | 1 |
|  | 74 | 1 | Reported Cases | 1 | 2 | 3 | 2 |
|  | 71 | 1 | Reported Cases | 1 | 2 | 1 | 1 |
|  | 70 | 1 | Reported Cases | 2 | 3 | 1 | 1 |
|  | 63 | 1 | Reported Cases | 1 | 1 | 1 | 1 |
|  | 45 | 2 | Reported Cases | 4 | 1 | 1 | 1 |
|  | 65 | 1 | Reported Cases | 2 | 1 | 3 | 2 |
|  | 69 | 2 | Reported Cases | 2 | 1 | 3 | 2 |
|  | 62 | 2 | Reported Cases | 4 | 1 | 2 | 1 |
|  | 75 | 1 | Reported Cases | 4 | 1 | 1 | 1 |
|  | 83 | 1 | Reported Cases | 2 | 1 | 2 | 2 |
|  | 72 | 1 | Reported Cases | 2 | 1 | 2 | 1 |
|  | 66 | 1 | Reported Cases | 2 | 1 | 2 | 1 |
|  | 77 | 1 | Reported Cases | 4 | 1 | 1 | 1 |
|  | 70 | 2 | Reported Cases | 4 | 1 | 1 | 1 |
|  | 74 | 1 | Reported Cases | 4 | 1 | 1 | 1 |
|  | 67 | 2 | Reported Cases | 4 | 1 | 2 | 2 |
|  | 66 | 2 | Reported Cases | 3 | 1 | 1 | 1 |
|  | 84 | 2 | Reported Cases | 3 | 1 | 2 | 2 |
|  | 78 | 1 | Reported Cases | 3 | 1 | 2 | 1 |
|  | 65 | 1 | Reported Cases | 1 | 1 | 1 | 1 |
|  | 77 | 2 | Reported Cases | 2 | 1 | 1 | 2 |
|  | 84 | 2 | Reported Cases | 4 | 1 | 2 | 1 |
|  | 65 | 1 | Reported Cases | 4 | 1 | 1 | 1 |
|  | 65 | 1 | Reported Cases | 2 | 1 | 2 | 1 |
|  | 77 | 1 | Reported Cases | 4 | 1 | 1 | 1 |
|  | 45 | 1 | Reported Cases | 2 | 1 | 1 | 1 |
|  | 78 | 1 | Reported Cases | 1 | 1 | 2 | 1 |
|  | 55 | 2 | Reported Cases | 1 | 1 | 2 | 1 |
|  | 72 | 1 | Reported Cases | 4 | 1 | 2 | 1 |
|  | 66 | 1 | Reported Cases | 4 | 1 | 1 | 1 |
|  | 83 | 1 | Reported Cases | 4 | 1 | 2 | 1 |
|  | 55 | 2 | Reported Cases | 1 | 1 | 1 | 1 |
|  | 75 | 1 | Reported Cases | 2 | 1 | 1 | 1 |
|  | 66 | 1 | Reported Cases | 1 | 1 | 1 | 1 |
|  | 55 | 2 | Reported Cases | 1 | 1 | 2 | 1 |
|  | 70 | 2 | Reported Cases | 2 | 1 | 1 | 1 |
|  | 79 | 2 | Reported Cases | 3 | 1 | 2 | 1 |
|  | 30 | 2 | Reported Cases | 4 | 1 | 1 | 1 |
|  | 72 | 2 | Reported Cases | 3 | 1 | 1 | 1 |
|  | 72 | 1 | Reported Cases | 3 | 1 | 1 | 1 |
|  | 86 | 1 | Reported Cases | 4 | 1 | 1 | 1 |
|  | 66 | 1 | Reported Cases | 2 | 1 | 3 | 2 |
|  | 48 | 2 | Reported Cases | 4 | 1 | 1 | 1 |
|  | 69 | 1 | Reported Cases | 2 | 1 | 1 | 1 |
|  | 67 | 1 | Reported Cases | 3 | 1 | 1 | 1 |
|  | 70 | 2 | Reported Cases | 1 | 2 | 2 | 1 |
|  | 78 | 1 | Reported Cases | 3 | 3 | 1 | 2 |
|  | 59 | 1 | Reported Cases | 3 | 3 | 1 | 1 |
|  | 78 | 2 | Reported Cases | 1 | 3 | 1 | 1 |
|  | 57 | 1 | Reported Cases | 2 | 3 | 2 | 1 |
|  | 55 | 1 | Reported Cases | 1 | 3 | 2 | 1 |
|  | 77 | 1 | Reported Cases | 2 | 3 | 1 | 1 |
