# Supplementary material for: Prognosis of immune checkpoint inhibitor-induced myasthenia gravis: a single center experience and systematic review
Source: Front Neurol. 2024 Apr 3;15:1372861. doi: 10.3389/fneur.2024.1372861 (PMC11022771; doi:10.3389/fneur.2024.1372861)
Supplement: Supplementary file 5 [file Table_5.DOCX]

Supplementary Table 5. ICI-related MG clinical manifestations, treatment and outcomes.

| Patient ID | Preexisting MG | Ptosis | Diplopia | Dysphasia | Dyspnea | Limb weakness | Anti-AChR | Other irAEs | Corticosteroids | Acetylcholinesterase inhibitors | IVIG | Plasmapheresis | ventilation | MG outcome | Death |
| --- | --- | --- | --- | --- | --- | --- | --- | --- | --- | --- | --- | --- | --- | --- | --- |
|  | 1 Yes, 2 No | 1 Yes, 2 No | 1 Yes, 2 No | 1 Yes, 2 No | 1 Yes, 2 No | 1 Yes, 2 No | 1 Positve, 2 Negative | 1 myositis,2 myocarditis,3 myositis+myocarditi | 1 Yes, 2 No | 1 Yes, 2 No | 1 Yes, 2 No | 1 Yes, 2 No | 1 Yes, 2 No | 1 Deterioration,2 Improvement,3 Complete resolution | 1 MG complications,2 Cancer progression,3 Other comorbidities |
| 1 | 2 | 1 | 1 | 2 | 2 | 1 | 1 | 2 | 1 | 2 | 2 | 1 | 2 | 2 | N |
| 2 | 2 | 2 | 2 | 2 | 2 | 1 | 2 | 2 | 2 | 2 | 1 | 2 | 2 | 2 | N |
| 3 | 2 | 1 | 1 | 2 | 2 | 1 | 2 | 3 | 1 | 2 | 2 | 2 | 2 | 2 | 2 |
| 4 | 2 | 1 | 2 | 2 | 2 | 2 | 1 | 2 | 2 | 2 | 2 | 2 | 2 | 2 | N |
| 5 | 2 | 1 | 2 | 2 | 2 | 2 | 2 | 2 | 2 | 2 | 2 | 2 | 2 | 2 | N |
| 6 | 2 | 2 | 2 | 2 | 2 | 1 | 2 | 3 | 2 | 2 | 1 | 2 | 2 | 1 | 2 |
| 7 | 2 | 2 | 2 | 2 | 2 | 2 | 2 | 1 | 2 | 2 | 2 | 2 | 2 | 2 | 2 |
| 8 | 2 | 1 | 1 | 2 | 2 | 1 | 2 | N | 2 | 2 | 2 | 2 | 2 | 2 | N |
| 9 | 2 | 2 | 2 | 2 | 2 | 2 | 2 | N | 1 | 1 | 1 | 2 | 2 | 2 | N |
| 10 | 2 | 2 | 1 | 1 | 2 | 2 | 2 | N | 1 | 2 | 2 | 2 | 2 | 2 | 2 |
| 11 | 2 | 1 | 2 | 2 | 2 | 2 | 2 | N | 1 | 2 | 2 | 2 | 2 | 3 | N |
| 12 | 2 | 1 | 2 | 1 | 2 | 2 | 2 | N | 1 | 2 | 2 | 2 | 2 | 2 | N |
| 13 | 2 | 2 | 2 | 2 | 2 | 2 | 1 | N | 1 | 2 | 2 | 2 | 2 | 1 | 1 |
| 14 | 2 | 1 | 2 | 2 | 1 | 2 | 2 | N | 1 | 1 | 2 | 1 | 2 | 1 | 2 |
| 15 | 2 | 1 | 2 | 1 | 2 | 1 | 1 | N | 1 | 1 | 1 | 2 | 2 | 2 | 3 |
| 16 | 2 | 1 | 2 | 2 | 2 | 2 | 2 | N | 2 | 1 | 2 | 2 | 2 | 3 | N |
| 17 | 2 | 1 | 2 | 2 | 2 | 1 | 1 | N | 1 | 2 | 2 | 2 | 2 | 2 | N |
| 18 | 2 | 2 | 2 | 1 | 1 | 2 | 1 | N | 2 | 1 | 1 | 2 | 1 | 2 | 2 |
| 19 | 2 | 1 | 2 | 2 | 2 | 2 | 2 | N | 1 | 1 | 1 | 2 | 2 | 3 | N |
| 20 | 1 | 1 | 2 | 2 | 2 | 1 | 1 | N | 1 | 2 | 1 | 2 | 1 | 2 | N |
| 21 | 1 | 2 | 1 | 2 | 2 | 2 | 1 | N | 1 | 2 | 2 | 2 | 2 | 2 | N |
| 22 | 1 | 1 | 1 | 2 | 2 | 2 | 1 | N | 1 | 1 | 1 | 1 | 1 | 2 | 3 |
| 23 | 1 | 2 | 2 | 1 | 1 | 2 | 2 | N | 1 | 2 | 1 | 1 | 2 | 2 | N |
| 24 | 2 | 1 | 1 | 2 | 2 | 2 | 1 | 1 | 1 | 1 | 1 | 2 | 2 | 2 | 3 |
| 25 | 2 | 2 | 2 | 2 | 1 | 1 | 1 | N | 1 | 1 | 1 | 1 | 2 | 2 | N |
| 26 | 1 | 1 | 1 | 1 | 2 | 1 | 1 | N | 1 | 1 | 1 | 1 | 2 | 1 | 1 |
| 27 | 2 | 1 | 1 | 2 | 2 | 2 | 2 | N | 1 | 1 | 1 | 1 | 2 | 2 | N |
| 28 | 2 | 1 | 1 | 2 | 2 | 1 | 2 | N | 1 | 1 | 1 | 2 | 2 | 2 | 3 |
| 29 | 2 | 2 | 2 | 2 | 1 | 2 | 2 | N | 1 | 1 | 1 | 2 | 1 | 2 | N |
| 30 | 1 | 2 | 1 | 2 | 1 | 2 | 1 | 1 | 1 | 1 | 2 | 1 | 2 | 1 | 1 |
| 31 | 2 | 1 | 1 | 1 | 2 | 2 | 2 | N | 1 | 1 | 2 | 2 | 2 | 2 | N |
| 32 | 2 | 1 | 1 | 2 | 1 | 2 | 1 | N | 2 | 1 | 2 | 2 | 2 | 2 | N |
| 33 | 2 | 1 | 1 | 1 | 2 | 1 | 1 | N | 1 | 1 | 1 | 2 | 2 | 2 | N |
| 34 | 2 | 2 | 2 | 2 | 2 | 2 | 2 | N | 1 | 1 | 1 | 2 | 2 | 2 | 2 |
| 35 | 2 | 1 | 1 | 2 | 2 | 2 | 1 | N | 1 | 1 | 1 | 2 | 2 | 2 | N |
| 36 | 2 | 2 | 2 | 1 | 2 | 2 | 1 | N | 1 | 1 | 1 | 2 | 2 | 2 | 3 |
| 37 | 2 | 1 | 2 | 2 | 2 | 2 | 1 | N | 1 | 1 | 2 | 2 | 2 | 3 | 3 |
| 38 | 2 | 1 | 1 | 2 | 2 | 2 | 2 | N | 2 | 1 | 2 | 2 | 2 | 2 | N |
| 39 | 1 | 2 | 1 | 2 | 2 | 2 | 1 | N | 1 | 1 | 2 | 1 | 1 | 1 | N |
| 40 | 2 | 1 | 1 | 1 | 2 | 2 | 1 | N | 1 | 2 | 1 | 1 | 1 | 2 | N |
| 41 | 2 | 2 | 1 | 1 | 2 | 1 | 2 | N | 1 | 2 | 2 | 2 | 2 | 2 | N |
| 42 | 2 | 1 | 2 | 2 | 2 | 1 | 2 | N | 1 | 1 | 2 | 1 | 2 | 3 | N |
| 43 | 2 | 1 | 2 | 2 | 1 | 2 | 2 | N | 1 | 1 | 1 | 2 | 2 | 2 | N |
| 44 | 2 | 1 | 1 | 2 | 2 | 2 | 1 | N | 1 | 2 | 1 | 2 | 2 | 2 | N |
| 45 | 2 | 1 | 2 | 1 | 2 | 2 | 2 | N | 1 | 1 | 1 | 2 | 2 | 2 | N |
| 46 | 2 | 1 | 1 | 2 | 1 | 1 | 2 | N | 1 | 2 | 2 | 1 | 2 | 2 | N |
| 47 | 2 | 1 | 1 | 2 | 2 | 1 | 1 | N | 1 | 1 | 2 | 2 | 2 | 2 | N |
| 48 | 2 | 1 | 1 | 1 | 2 | 1 | 1 | N | 1 | 1 | 2 | 2 | 2 | 2 | N |
| 49 | 2 | 1 | 1 | 1 | 1 | 1 | 2 | N | 1 | 1 | 2 | 2 | 1 | 2 | N |
| 50 | 1 | 1 | 2 | 1 | 2 | 2 | 1 | N | 1 | 1 | 1 | 1 | 2 | 2 | 3 |
| 51 | 2 | 2 | 1 | 1 | 2 | 2 | 2 | N | 1 | 2 | 2 | 2 | 2 | 2 | N |
| 52 | 2 | 2 | 1 | 2 | 2 | 2 | 1 | N | 2 | 1 | 2 | 2 | 2 | 2 | 2 |
| 53 | 2 | 2 | 1 | 2 | 1 | 2 | 1 | N | 1 | 1 | 1 | 2 | 2 | 2 | 3 |
| 54 | 2 | 2 | 1 | 2 | 2 | 2 | 2 | N | 1 | 1 | 1 | 1 | 1 | 1 | 1 |
| 55 | 2 | 2 | 2 | 1 | 1 | 1 | 2 | N | 1 | 1 | 1 | 2 | 2 | 2 | N |
| 56 | 2 | 1 | 2 | 2 | 2 | 2 | 2 | N | 1 | 1 | 1 | 1 | 2 | 2 | N |
| 57 | 2 | 1 | 1 | 1 | 2 | 2 | 1 | N | 1 | 1 | 2 | 1 | 2 | 2 | N |
| 58 | 2 | 2 | 2 | 2 | 1 | 1 | 1 | N | 1 | 1 | 2 | 2 | 2 | 2 | 2 |
| 59 | 2 | 2 | 1 | 2 | 1 | 1 | 2 | N | 1 | 1 | 2 | 2 | 2 | 2 | N |
| 60 | 1 | 2 | 2 | 2 | 1 | 1 | 1 | N | 1 | 1 | 1 | 1 | 2 | 1 | 1 |
| 61 | 2 | 1 | 1 | 2 | 2 | 2 | 1 | N | 1 | 2 | 1 | 1 | 1 | 1 | 1 |
| 62 | 2 | 1 | 2 | 2 | 1 | 2 | 1 | 1 | 1 | 1 | 1 | 1 | 1 | 1 | 1 |
| 63 | 2 | 1 | 2 | 2 | 2 | 2 | 1 | 2 | 1 | 2 | 1 | 1 | 1 | 1 | 1 |
| 64 | 2 | 1 | 1 | 2 | 2 | 2 | 2 | 1 | 1 | 1 | 2 | 2 | 2 | 1 | 1 |
| 65 | 2 | 2 | 1 | 2 | 2 | 2 | 1 | 2 | 1 | 2 | 2 | 2 | 1 | 2 | N |
| 66 | 1 | 2 | 2 | 2 | 2 | 1 | 1 | 1 | 1 | 2 | 2 | 2 | 2 | 2 | N |
| 67 | 2 | 1 | 2 | 2 | 2 | 2 | 1 | 1 | 1 | 1 | 2 | 1 | 1 | 1 | 3 |
| 68 | 2 | 1 | 1 | 2 | 2 | 2 | 2 | 1 | 1 | 1 | 2 | 2 | 2 | 3 | N |
| 69 | 2 | 2 | 1 | 2 | 1 | 2 | 1 | 1 | 1 | 2 | 2 | 1 | 1 | 1 | N |
| 70 | 2 | 2 | 2 | 2 | 2 | 2 | 1 | 3 | 1 | 1 | 1 | 2 | 1 | 2 | N |
| 71 | 2 | 1 | 2 | 2 | 1 | 2 | 1 | 2 | 1 | 2 | 2 | 2 | 2 | 2 | N |
| 72 | 2 | 2 | 2 | 2 | 1 | 2 | 1 | 2 | 1 | 1 | 2 | 1 | 1 | 2 | N |
| 73 | 2 | 2 | 2 | 2 | 1 | 2 | 1 | 1 | 1 | 2 | 1 | 2 | 1 | 1 | 1 |
| 74 | 2 | 1 | 2 | 2 | 2 | 1 | 1 | 2 | 1 | 2 | 1 | 2 | 2 | 2 | 3 |
| 75 | 2 | 1 | 2 | 2 | 2 | 1 | 2 | 2 | 1 | 1 | 2 | 2 | 2 | 1 | 1 |
| 76 | 2 | 1 | 1 | 2 | 2 | 2 | 2 | 1 | 1 | 2 | 1 | 1 | 2 | 1 | 1 |
| 77 | 2 | 2 | 2 | 2 | 2 | 2 | 1 | 1 | 1 | 2 | 2 | 2 | 2 | 1 | 1 |
| 78 | 2 | 2 | 1 | 2 | 2 | 2 | 1 | 3 | 1 | 1 | 2 | 2 | 1 | 1 | 1 |
| 79 | 2 | 1 | 2 | 2 | 2 | 2 | 1 | 1 | 1 | 2 | 2 | 1 | 1 | 2 | N |
| 80 | 2 | 1 | 2 | 2 | 2 | 1 | 2 | 1 | 1 | 1 | 2 | 2 | 2 | 2 | N |
| 81 | 2 | 1 | 2 | 2 | 2 | 1 | 1 | 2 | 1 | 2 | 1 | 2 | 1 | 2 | N |
| 82 | 2 | 1 | 1 | 2 | 2 | 2 | 2 | 1 | 1 | 1 | 1 | 2 | 2 | 1 | 1 |
| 83 | 2 | 1 | 1 | 2 | 2 | 2 | 1 | 2 | 1 | 2 | 1 | 2 | 2 | 1 | 1 |
| 84 | 2 | 1 | 2 | 2 | 1 | 2 | 1 | 1 | 1 | 1 | 1 | 2 | 1 | 2 | N |
| 85 | 2 | 1 | 2 | 2 | 2 | 1 | 1 | 2 | 1 | 1 | 1 | 1 | 1 | 2 | N |
| 86 | 2 | 1 | 2 | 1 | 1 | 1 | 1 | 2 | 1 | 2 | 1 | 1 | 1 | 2 | N |
| 87 | 1 | 2 | 1 | 1 | 1 | 1 | 1 | 3 | 1 | 2 | 1 | 2 | 2 | 1 | 1 |
| 88 | 2 | 1 | 2 | 1 | 1 | 1 | 1 | 2 | 1 | 2 | 1 | 1 | 2 | 1 | 1 |
| 89 | 2 | 2 | 2 | 1 | 2 | 2 | 1 | 1 | 1 | 2 | 1 | 1 | 1 | 2 | 2 |
| 90 | 2 | 2 | 1 | 2 | 2 | 1 | 1 | 2 | 1 | 1 | 1 | 1 | 1 | 2 | N |
| 91 | 2 | 1 | 2 | 1 | 2 | 1 | 1 | 1 | 1 | 1 | 1 | 2 | 1 | 1 | 1 |
| 92 | 2 | 1 | 2 | 2 | 1 | 2 | 1 | 2 | 1 | 2 | 1 | 1 | 1 | 2 | N |
| 93 | 1 | 2 | 2 | 1 | 2 | 2 | 1 | 3 | 1 | 2 | 1 | 1 | 1 | 2 | N |
| 94 | 2 | 1 | 2 | 2 | 1 | 2 | 1 | 3 | 1 | 1 | 1 | 2 | 1 | 1 | 1 |
| 95 | 2 | 1 | 2 | 2 | 2 | 2 | 2 | 1 | 2 | 2 | 2 | 2 | 2 | 3 | N |
| 96 | 2 | 1 | 1 | 2 | 2 | 1 | 1 | 3 | 1 | 1 | 1 | 2 | 1 | 2 | 3 |
| 97 | 2 | 2 | 2 | 2 | 2 | 1 | 1 | 2 | 1 | 2 | 1 | 2 | 2 | 2 | 1 |
| 98 | 2 | 2 | 2 | 2 | 2 | 1 | 1 | 2 | 1 | 2 | 1 | 2 | 2 | 1 | 1 |
| 99 | 2 | 1 | 2 | 2 | 2 | 1 | 1 | 3 | 1 | 2 | 1 | 1 | 2 | 1 | 1 |
| 100 | 2 | 2 | 1 | 2 | 1 | 2 | 2 | 1 | 1 | 1 | 1 | 2 | 2 | 2 | N |
| 101 | 2 | 2 | 2 | 2 | 1 | 2 | 1 | 2 | 1 | 1 | 2 | 2 | 2 | 1 | 1 |
| 102 | 2 | 1 | 2 | 2 | 1 | 2 | 1 | 2 | 1 | 2 | 1 | 2 | 1 | 2 | N |
| 103 | 2 | 1 | 1 | 1 | 2 | 2 | 1 | 2 | 1 | 2 | 1 | 2 | 2 | 1 | 1 |
| 104 | 2 | 2 | 2 | 1 | 2 | 2 | 1 | N | 1 | 1 | 2 | 1 | 2 | 2 | N |
| 105 | 2 | 1 | 1 | 1 | 2 | 1 | 2 | 1 | 1 | 2 | 2 | 1 | 2 | 2 | N |
| 106 | 2 | 1 | 2 | 2 | 2 | 2 | 1 | 2 | 1 | 2 | 1 | 1 | 2 | 2 | N |
| 107 | 2 | 2 | 1 | 2 | 2 | 1 | 2 | 3 | 1 | 2 | 1 | 1 | 2 | 1 | N |
| 108 | 2 | 1 | 2 | 2 | 1 | 1 | 1 | 1 | 1 | 1 | 2 | 2 | 2 | 2 | 3 |
| 109 | 2 | 1 | 2 | 2 | 2 | 2 | 2 | 2 | 1 | 2 | 1 | 1 | 2 | 2 | N |
| 110 | 2 | 1 | 1 | 2 | 2 | 2 | 1 | 2 | 1 | 2 | 2 | 2 | 1 | 2 | N |
